# Spatial ecology of cane toads (*Rhinella marina*) in their native range: a radiotelemetric study from French Guiana

**DOI:** 10.1038/s41598-021-91262-8

**Published:** 2021-06-03

**Authors:** Jayna L. DeVore, Richard Shine, Simon Ducatez

**Affiliations:** 1 School of Life and Environmental Sciences, The University of Sydney, Sydney, NSW 2006 Australia; 2 Department of Biological Sciences, Macquarie University, Sydney, NSW 2109 Australia; 3 Institut de Recherche Pour Le Développement (IRD), UMR 241 EIO (UPF, IRD, IFREMER, ILM), Papeete, Tahiti French Polynesia

**Keywords:** Evolutionary ecology, Forest ecology, Freshwater ecology, Invasive species, Riparian ecology, Tropical ecology, Wetlands ecology

## Abstract

Like most invasive species, cane toads have attracted less research in their native range than in invaded areas. We radio-tracked 34 free-ranging toads in French Guiana, a source region for most invasive populations, across two coastal and two rainforest sites. Coastal toads generally sheltered in pools of fresh or brackish water but nocturnally foraged on beaches, whereas rainforest toads sheltered in forested habitats, moving into open areas at night. Over five days of monitoring, native toads frequently re-used shelters and moved little between days (means = 10–63 m/site) compared to invasion-front toads from Australia (~ 250 m). Larger toads moved less between days, but displaced in more consistent directions. At night, foraging toads travelled up to 200 m before returning to shelters. Foraging distance was related to body condition at coastal sites, with toads in poorer body condition travelling farther. Rain increased the probability of coastal toads sheltering in the dry habitats where they foraged. Dispersal and rainfall were lower at coastal sites, and the strategies utilized by coastal toads to minimize water loss resembled those of invasive toads in semi-desert habitats. This global invader already exhibits a broad environmental niche and substantial behavioural flexibility within its native range.

## Introduction

The ecological and economic impacts of invasive species can be massive^1^, stimulating extensive scientific research on the biology of invaders^2–4^. Unsurprisingly, most of that research has been conducted in places where the species involved causes problems—that is, in regions that have been invaded—rather than in the species’ native range^5^. The majority of research on the biology of “pest species” in their countries of origin has been to identify natural enemies (parasites, pathogens, etc.) that might be used for biocontrol in the invaded regions^6^. As a result, most of what we know about the biological attributes of invasive species tends to be derived from the invaded rather than native range of those taxa.

That geographic focus holds true for cane toads (*Rhinella marina*), a large bufonid anuran native to South America but translocated to many countries around the world in a futile attempt at biocontrol of insect pests in commercial plantations^7,8^. This toxic toad’s devastating impact on Australian predators has stimulated hundreds of studies, including several radiotelemetry-based projects on the movement patterns of free-ranging toads within Australia^9–26^ and one study on invasive populations in Hawai’i^27^. In combination, that work has documented strong geographic variation in spatial ecology within the toads’ extensive introduced range. In particular, the extremely high dispersal abilities of Australian cane toads from invasion-front populations relative to long-established populations have highlighted how the rapid evolution of dispersal abilities can accelerate the invasion process^15–17,21^. However, such a high level of variation among populations makes it difficult to clarify the ancestral condition of movement patterns for the species. Toads were serially translocated from their native range in the Guianas, to the Caribbean, then to Hawai’i (in 1932) before being brought to Australia in 1935^7^. Thus, the attributes of modern-day Hawai’ian toads^27^ may reflect adaptations to island life rather than traits present in the original colonists from South America, much as dispersal rates in Australia have evolved during the invasion of the Australian continent. As a result, the best way to identify the likely ancestral condition for spatial ecology of invasive populations of cane toads is to study the species in its native range—preferably, the region from which toads were taken for those international translocations. We have conducted such a study.

## Methods

### Study species

*Rhinella marina* (cane toad, marine toad) is a large bufonid anuran species that has been introduced to > 100 countries and islands worldwide^7^. All of the introduced populations of this global invader across the Caribbean and Pacific were originally sourced from the Guianas (with the exception of Florida, where toads from Colombia were also introduced)^7^, and mitochondrial studies identify French Guiana as the source of the toads that were brought to Australia via Hawai’i and Puerto Rico^28^. The native range of this species is in areas east of the Andes in South America; west-of-Andes populations and Central American populations previously referred to this species have now been allocated to a separate taxon, *R. horribilis*^29^. Cane toads are generally diurnal during postmetamorphic life, but shift to primarily nocturnal activity as they grow larger^30^. They typically spend the day inside damp terrestrial retreat-sites, the use of which reduces water loss^31,32^, but emerge at night to forage for prey (mostly small insects, but occasionally spiders, small vertebrates, etc.^33^). Toads lack specialized physiological adaptations for reducing water loss in arid environments, but use behavioural mechanisms to maintain moisture^34^; in dry weather cane toads need to rehydrate frequently and usually do so by lying in pools or on damp ground^35^. Although the permeable skin of amphibians also renders them sensitive to osmotically stressful environments, cane toads have physiological strategies for regulating salts and are relatively tolerant of saltwater^36^. In seasonally arid regions cane toads are active primarily during wetter times of year, but movement and breeding occurs year-round in suitably warm damp habitats^37,38^. Toads generally avoid breeding in waterbodies surrounded by dense vegetation, instead preferentially breeding in temporary pools in open habitats^39^.

### Study sites

**Figure 1:**
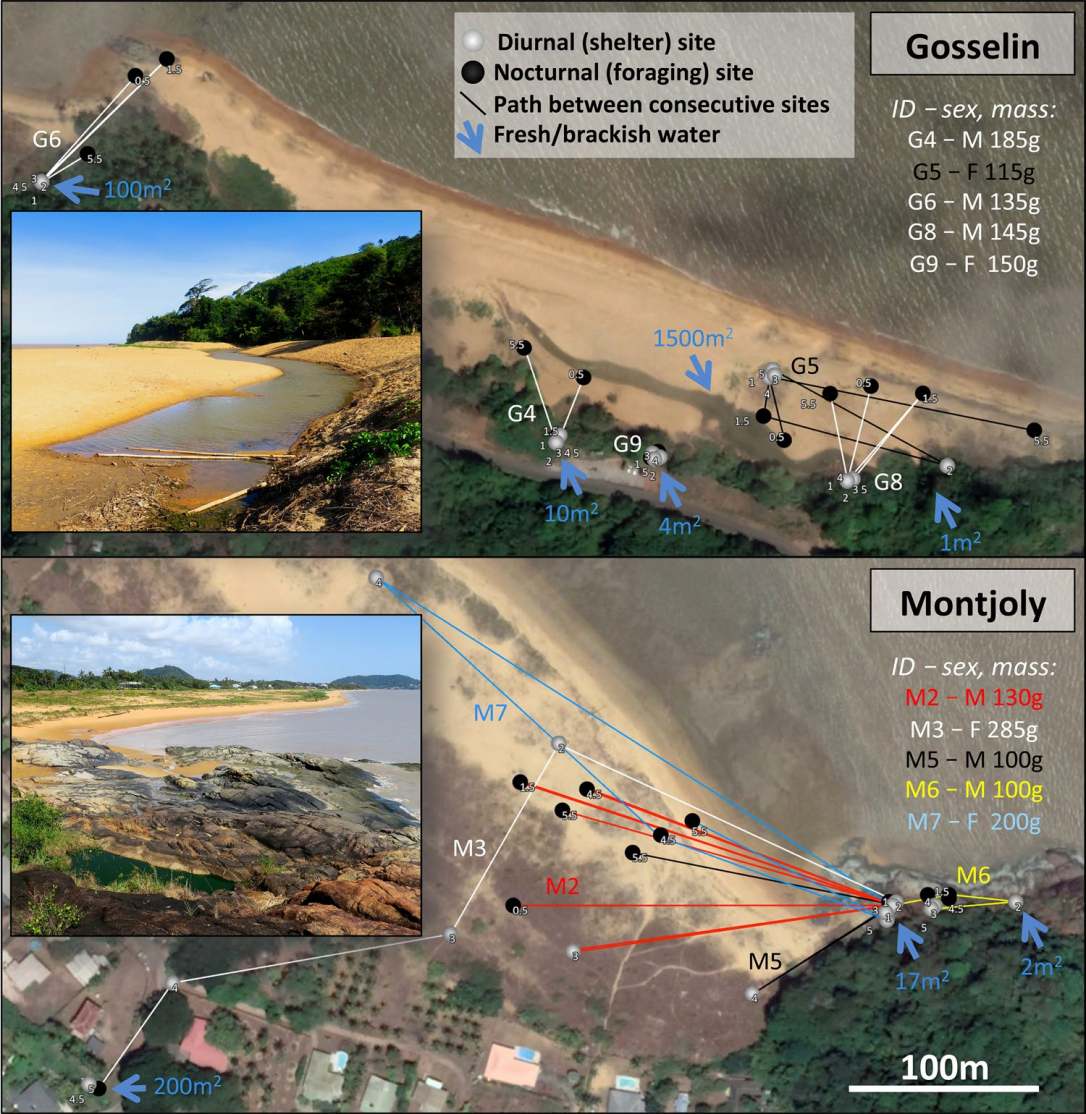
Coastal sites at which cane toads were radio-tracked, showing general features and paths followed by 10 of the 20 radio-tracked animals. Small numbers adjacent to each point indicate temporal sequences. See Supplementary Materials for extended version of this caption. Photo credit: J. DeVore, with satellite imagery from Google Earth.

**Figure 2:**
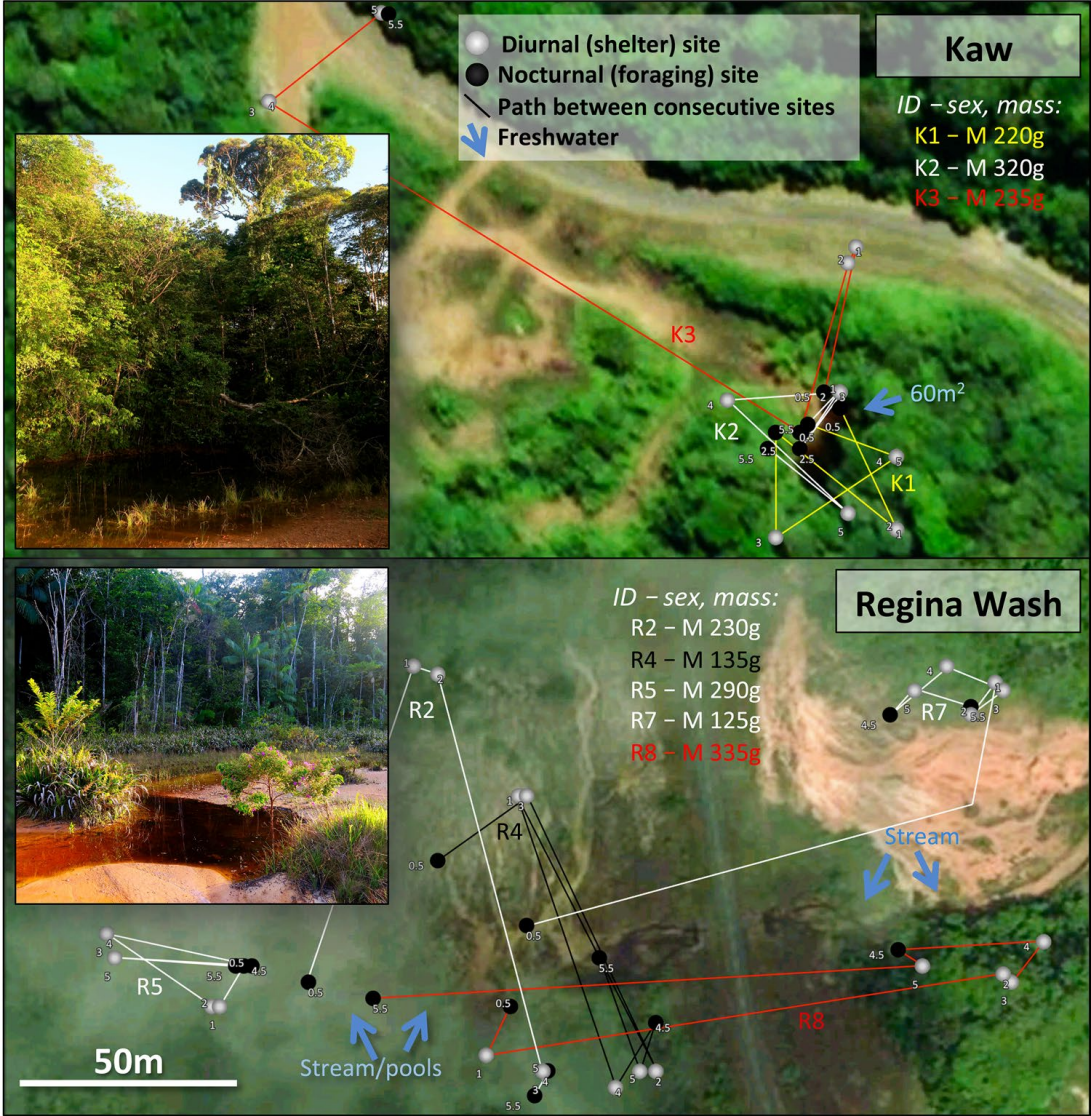
Rainforest sites at which cane toads were radio-tracked, showing general features and paths followed by 8 of the 14 tracked animals. Small numbers adjacent to each point indicate temporal sequences. See Supplementary Materials for extended version of this caption. Photo credit: J. DeVore, with satellite imagery from Apple Maps.

In French Guiana > 95% of the land area is forested, but cane toads occupy a range of habitat types from dense rainforest to coastal beaches^40^. To include that variation, we tracked toads at two sites in coastal beach habitats (Fig. 1) and two sites dominated by tropical rainforest (Fig. 2). The climate in French Guiana is hot and humid year-round, with mean air temperature > 25 °C in all months and an average monthly rainfall of > 70 mm year-round and > 250 mm from December to June^41^ (Supplementary Fig. S1). Our telemetry was conducted in August (Gosselin, Montjoly, Kaw) and September (Regina Wash) 2017. These sites are described briefly below, with additional details available in the Supplementary Methods.

#### Gosselin Beach (4.8907S, − 52.2529 W, altitude 5 m asl)

Gosselin is a sandy beach in Rémire-Montjoly backed by a narrow band of forest. This site contains a 1500 m^2^ pool surrounded by sand and, along one edge, tall, dense grass. A second pool ~ 250 m to the east was also used by toads, as were other, smaller sources of fresh or brackish water (a roadside pool, a seep, and a shallow puddle below a rock outcrop). Six male toads and four female toads were radio-tracked at this site.

#### Montjoly Beach (4.9133S, − 52.2599 W, altitude 5 m asl)

The eastern edge of this beach (~ 3 km N of Gosselin) abuts a rock outcrop with ephemeral pools, the largest of which is a 17 m^2^ rock pool 50 m from the beach. Many toads (males, females, and juveniles) were found gathered around this pool (frequently the only waterbody in the area; Supplementary Fig. S2 online). Four male toads and six female toads were radio-tracked at this site.

#### Kaw Rainforest (4.6437S, − 52.2991 W, altitude 240 m asl)

This tropical rainforest site is ~ 28 km S of Gosselin and surrounds a permanent pond in the Kaw forest. Three male toads and one female were radio-tracked at this site.

#### Regina Wash Rainforest (4.2944S, − 52.2205 W, altitude 45 m asl)

This open wash (~ 500 m^2^) within the tropical rainforest is located ~ 30 km S of the Kaw site and surrounds a slowly flowing stream. Ten male toads were radio-tracked at this site. Only one female was observed at this site, and this occurred after the tracking period.

### Radio-tracking

**Figure 3:**
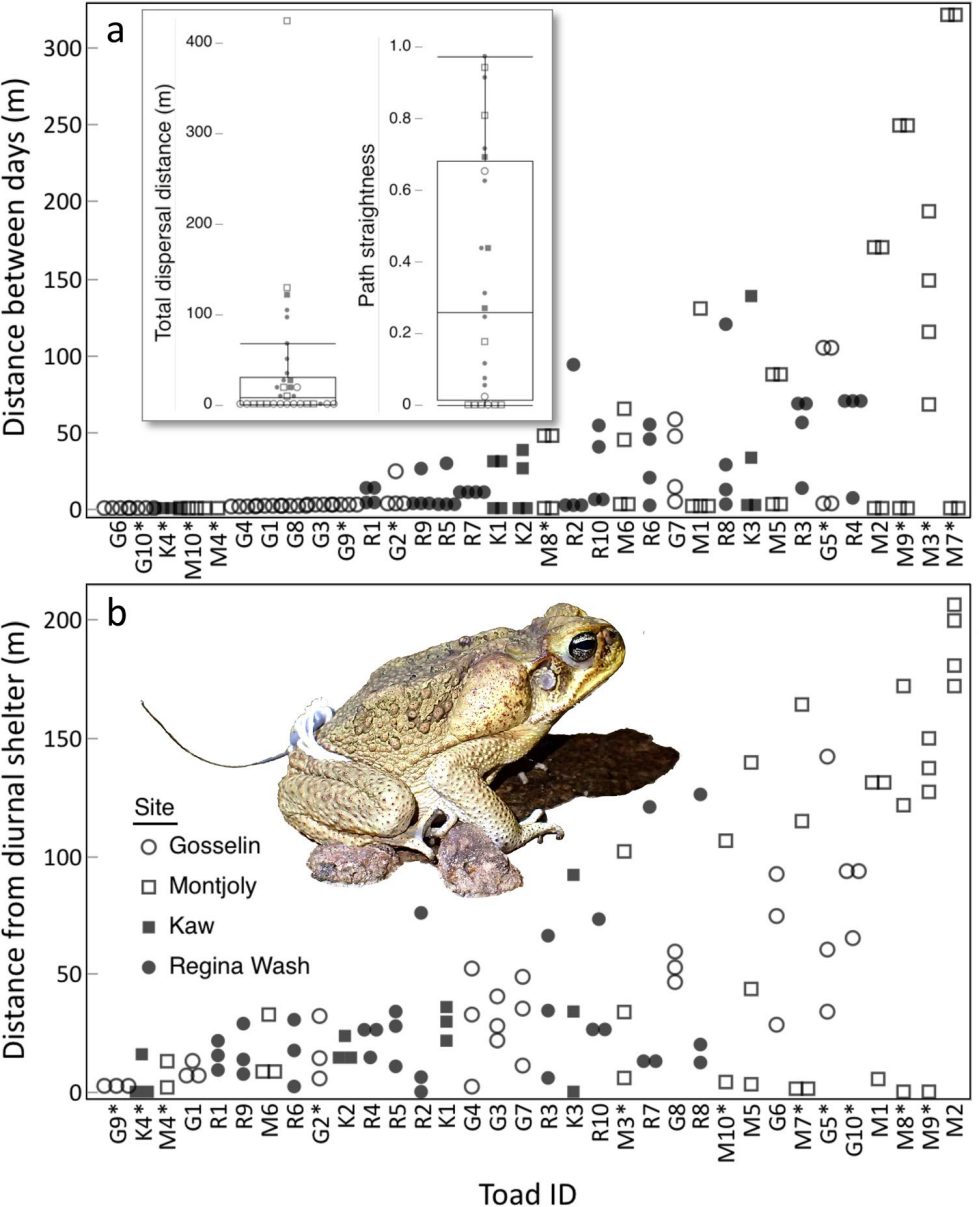
Movements of individual cane toads, ordered by ascending mean values: (**a**) the distance each toad moved between successive days (4 values/toad, except 1 for M4), (**b**) the distance toads travelled from their diurnal shelters at night (3–4 values/toad, except 2 for M4 and M10). The panel (**a**) inset boxplots indicate the total distance moved over the 5-day tracking period (mean: 37 m), as well as how straight this movement was (mean straightness 0.35; a path straightness value of 0 would indicate that the toad had returned to its original shelter at the end of the tracking period, whereas a value of 1 would indicate that its movements between diurnal shelters were linear, consistently taking it farther from its original shelter). Plots depict the medians and interquartile ranges, with whiskers designating the minimum and maximum non-outlier values. Asterisked toad IDs identify females. See Supplementary Materials for extended version of this caption. Photo credit: J. DeVore.

We captured 34 adult toads across the four sites, sexed, and weighed them. Sex was determined from morphology (toe pads, skin rugosity) and the male-specific “release call”^42^. We attached radio-transmitters (Holohil PD-2; ~ 3.5 g, < 4% of toad mass) to cotton twine waist-belts, and re-released each animal at its point of capture within 10 min of capture. The majority of toads (26) were initially captured at night. However, we also sought to determine whether nocturnal collection biased our movement estimates towards active individuals. We therefore also located eight toads within their diurnal shelters and attached their radio-transmitters during the day. Locating new toads during the day was not possible at the rainforest sites, where toads were dispersed throughout the forest, so seven of the eight “diurnal start” toads were from coastal habitats. The only “diurnal start” toad from the rainforest was the only rainforest female ever observed during the tracking period; a large 690 g (210 mm SVL) toad discovered while searching pondside burrows for one of the tracked male toads. However, this female was missing a leg (a common injury, especially in beach toads). As this could have biased her movements, we describe these movements in Fig. 3 (individual K4), but excluded this individual from all analyses. Toad selection at coastal sites was haphazard (though we attempted to track an even balance of males and females and avoided individuals that were emaciated or had missing limbs), whereas at the rainforest sites we tracked nearly all of the adult toads found on either Night 1 or Day 1. Morphologically, coastal toads were smaller than rainforest toads and those at Gosselin tended to be fatter (Supplementary Fig. S3 online).

After equipping them with radio-transmitters, we located each animal daily (in its diurnal retreat-site), at which time we took a GPS reading each day for 5 days. During that period, the toads were also located on 3 nights (when toads typically emerge to forage). Our estimates of how far toads travelled from their diurnal shelter during their nocturnal expeditions are therefore conservative, as they are based on a single point per night. Sunset occurred at 1840 h during the tracking period, and toads typically began to emerge at 1900 h. The nocturnal locations of each toad were then taken between 2030 and 0130 h. At the end of the 5-day period, the toads were recaptured, and their radio-transmitters were removed. To determine whether body size had influenced toad movements we also measured SVL at this time. Rain can also affect toad movements^19^, so we noted whether or not it had rained in the 24 h preceding each capture event. Toad positions in the absence and presence of recent rain were not available at Gosselin, as it did not rain at Gosselin during the tracking period, but it did rain at all other sites (Montjoly rain: Day 4; Kaw rain: Days 4, 5; Regina Wash rain: Days 3, 4, 5, Nights 4, 5).

Behaviour can also be state-dependent; for example, body condition can influence foraging decisions^43^. To calculate the body condition of the tracked toads we obtained body mass (g) and SVL (mm) data from 142 male and 98 female toads from across northern French Guiana^40^. Using these data we calculated sex-specific relationships between mass and SVL by plotting ln(mass) against ln(SVL). For males this relationship was y = 2.868x − 8.739 (Pearson r = 0.9692), and for females it was y = 2.985x − 9.292 (Pearson r = 0.9696). We then used these values to calculate the scaled mass index for each tracked toad^44^. This index uses the formula *M*_i_ * (*L*_0_/*L*_i_)^*b*_SMA_, to compute the mass each toad would have if they all had the same body length (*L*_0_). Here, *M*_i_ and *L*_i_ are the mass and SVL of the individual, *L*_0_ was taken to be the mean SVL of the tracked toads (120 mm), and *b*_SMA_ is the slope of the sex-specific ln–ln regression of mass by SVL divided by the Pearson correlation coefficient (males 2.959, females 3.079).

### Descriptors of movements between diurnal shelter sites

We used each toad’s position within its daily refugia to calculate the following seven parameters:

(i) *Distance between days (continuous, m).* The distance between a toad’s position and its position on the previous day (4 values/toad).
(ii) *Probability of changing shelters (binomial, moved yes/no).* Whether or not a toad had moved > 5 m from the shelter it used the day before (4 values/toad).
(iii) *Distance between shelters (continuous, m).* If a toad had left its previous diurnal shelter, the distance between the old shelter and the new one (this measure is the same as distance between days, but excludes occasions when toads spent consecutive days at the same shelter site; ≤ 4 values/toad).
(iv) *Number of diurnal shelters used (integer between 1 and 5).* Toads sometimes left a diurnal shelter for a new one, only to return at a later date; we therefore also quantified the total number of diurnal shelters used by each toad over the 5-day period, using a 5 m cut-off to determine whether the toad had returned to the same shelter (1 value/toad).
(v) *Total dispersal distance (continuous, m).* The distance between the toad’s original shelter site on Day 1 and its diurnal shelter on Day 5 (1 value/toad).
(vi) *Path straightness (proportion).* Calculated as the ‘total dispersal distance’ divided by the sum of the distances between days (i.e., the proportion of the toad’s total movement that took it farther from its original location^45^; 1 value/toad); this proportion was logit transformed prior to analysis.
(vii) *Shelter type (binomial, water present/absent).* Each shelter site was categorized according to whether or not standing water was present; at shelter sites where water was classified as present, the toad was either sheltering in the water or within 5 m of it (5 values/toad). This variable was created to account for the possibility that the importance of standing water could vary in response to rain or between coastal and rainforest sites. For example, potential effects of rain on toad movements could have been obscured by the fact that, if a toad moved away from a shelter adjacent to standing water during rain, it might subsequently move back after the rain stopped (i.e., move an equal distance on a rainless day; such paired distances are evident in a number of Montjoly toads; Fig. 3). This variable was created to detect such potential shifts in shelter preference.

### Foraging movements

We used data on the nocturnal position of each toad to calculate:

(i) *Distance from diurnal shelter (continuous, m).* The distance between the toad’s diurnal refugium and its nocturnal location; where the location of the refugia used before and after that night were both known, we took the mean of these two distances (3 values/toad).
(ii) *Probability of foraging (binomial, foraged yes/no).* Whether or not the toad had emerged from its diurnal shelter to forage (i.e., was found > 5 m from its daytime refugium; 3 values/toad).
(iii) *Foraging distance (continuous, m)*. If a toad had emerged from its diurnal shelter, the distance between the diurnal shelter and where the animal was found foraging (this measure is the same as distance from diurnal shelter, but excludes occasions when toads did not emerge to forage; ≤ 3 values/toad).

Note that we refer to nocturnal movements as “foraging movements” for simplicity. However, breeding activity occurs year-round in cane toads (although it peaks in the wet season). Therefore, breeding activity could also have contributed to these movements. Although tadpoles were present and breeding occurs at all of the study sites, we never observed breeding or new egg clutches during the tracking period (though calling did occur).

### Exceptions

Two toads at Montjoly lost their transmitters before the end of the tracking period; a female (M10; 115 g) that dropped her transmitter on the final day, such that we obtained 5 days and 2 nights of data, and a female (M4; 105 g) for which we only obtained 2 days and 2 nights of data. This latter female (M4) was excluded from analyses of the number of diurnal shelters used, total dispersal distance, and path straightness. Another female toad entered private property on the final day of the tracking period, and we were only able to estimate her position within 10 m for Day 5 (M3; see Fig. 1). To minimize handling of the toads prior to tracking their movements, SVL measurements were taken at the conclusion of tracking; we therefore lack data on size and body condition for these three toads. We also tracked three of the other Montjoly toads for an additional night, yielding 5 days and 4 nights of data for these individuals. Finally, eight toads that used the same diurnal shelter every day (i.e., never moved > 5 m) were excluded from measures of path straightness, leaving 24 toads for this measure (3 Gosselin, 8 Montjoly, 3 Kaw, 10 Regina Wash). All available data were used for all analyses.

### Ethics declaration

All procedures were reported in accordance with ARRIVE guidelines, carried out in accordance with all relevant guidelines and regulations, and approved by the University of Sydney Animal Care and Ethics Committee (Approval 6033).

## Data analyses

### Effects of initial capture method, site, rainfall, and variation between individuals on toad movements

We first used data from the coastal toads to determine whether initially capturing toads at night (rather than during the day) biased our movement estimates. We therefore used separate models to analyse all of the movement parameters outlined above as responses to the fixed effect of initial capture method. To account for variation between sites and individuals, site and individual ID were included in these models as nested random effects. Upon finding that locating toads at night did not have pronounced effects on our movement estimates, we excluded this factor from all subsequent models.

We then used data from all of the sites (and toads from both initial capture methodologies) to test for site-specific differences in toad movement or shelter selection. For these models, each parameter outlined above was analysed as a response to the fixed effect of site. Because toads are nocturnally active, their nocturnal position might vary depending on when they were captured. To control for this factor, we also included time in analyses of nocturnal position (although toads were located in a randomized order, and our conclusions were not affected if time was omitted). Rain could also affect toad movements, or have effects that vary between sites. We therefore re-ran all of these models, but with the added predictor of whether or not it had rained in the previous 24 h (categorical: rain present/absent), as well as the interaction between site and rainfall.

To determine whether variation between individuals affected toad movements as well as whether the effects of individual variation differed between sites, we retained the fixed effect of site in each model (and time, for nocturnal positions) and added individual-level predictors to each model. These individual-level predictors included SVL (continuous: mm), body condition (continuous: scaled mass index, g), and sex (categorical: female, male). We also included all first-order interactions. Because all of the toads tracked in the rainforest were males, we first excluded the rainforest sites from all models and looked for an effect of these individual-level predictors on toad movement within coastal sites alone. For models where a significant effect of sex was found, we retained sex as a predictor in subsequent analyses in which data from all four sites were included. However, if no effect of sex was found within coastal sites, we omitted sex as a predictor in subsequent analyses of the full dataset. This was done to avoid confounding any effects of sex with those of site. (Within coastal sites, the only movement parameter that was significantly affected by sex was the probability that a toad would emerge to forage; therefore, this is the only model that included sex as a predictor when the full dataset was analysed).

### Statistical procedures

All analyses were conducted in R^46^. In all models, distances were ln-transformed (ln[y + 1]) prior to analysis to meet homoscedasticity assumptions. Any continuous response variables for which multiple measures were taken per toad were analysed as mixed-effect models (package nlme:lme^47^). Individual ID was included in these models as a random effect to account for variation between individual toads. Continuous measures for which there was only one final value per toad were instead analysed via linear regression using the lm procedure. Binomial responses were analysed via logistic regression (package lme4:glmer^48^), with individual ID included as a random effect. The number of diurnal shelters used per toad was analysed using a quasipoisson model (glm; to account for data underdispersion). For models that included multiple predictors (e.g., site as well as rain or individual-level predictors), we used a stepwise removal procedure to eliminate non-significant predictors from the model, starting with non-significant interactions. We used *p* = 0.1 as the threshold for elimination; therefore, the final models only included variables for which *p* < 0.1 (though non-significant main effects were retained if they were involved in a significant interaction). All models were initially assessed using the anova function (or package car:Anova^49^, for models with a binomial or Poisson distribution). Where significant effects were identified we used Tukey post-hoc tests (package emmeans^50^) to conduct pairwise comparisons and identify significant differences.

## Results

At all four sites our radio-tracked toads typically remained within shelter-sites by day, and moved at night. The spatial ecology of these toads varied among sites (Figs. 1,  2). These movements are described in Fig. 3.

### Effects of initial capture methodology on movement estimates

Coastal toads that were initially located at night were not significantly more likely to emerge to forage than were those initially captured during the day (df = 17, t = 1.43, *p* = 0.172), nor did night-captured toads move significantly farther from their diurnal shelters during the night than day-captured toads (effect size = 0.99 m, standard error [SE] = 0.28 to 2.10 m, df = 17, t = 1.56, *p* = 0.136). Indeed, the only significant difference between toads captured during the day and those captured at night was that those captured at night moved farther between diurnal shelters (effect size = 3.63 m, SE = 2.94 to 4.55 m, df = 8, t = 3.21, *p* = 0.0125). As toads that sheltered in the sand moved more between diurnal shelters (Fig. 1) but were also harder to locate during the day than were toads that sheltered together in standing water, this difference likely indicates that selecting toads during the day biased against these mobile, spatially-segregated individuals. As initial capture method did not strongly bias our movement estimates, this factor was excluded from all subsequent models.

### Differences in toad movement between sites

**Figure 4:**
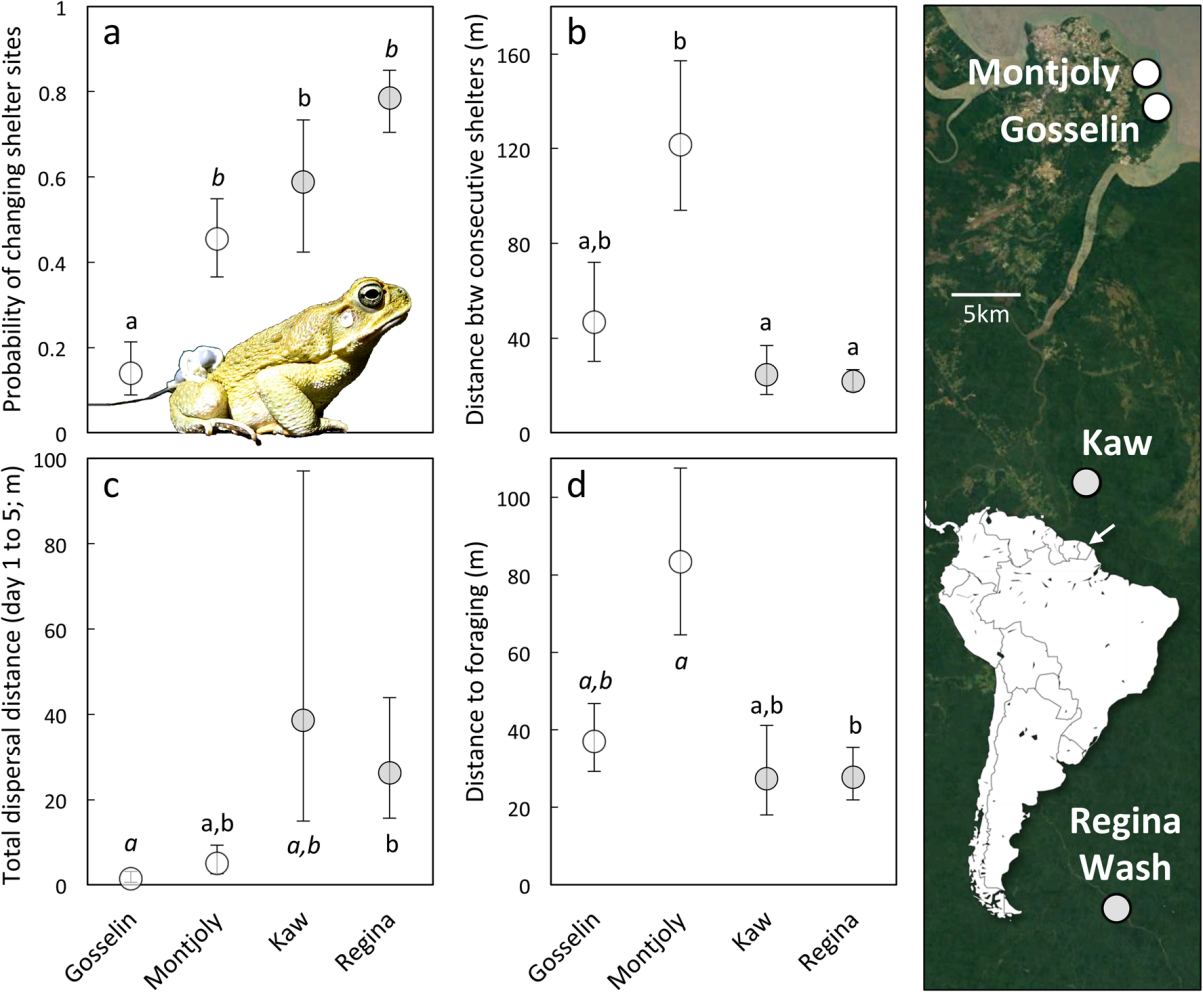
Cane toad movements at the four tracking sites, showing (**a**) probability of changing shelter-sites, (**b**) distance between consecutive shelters, (**c**) total dispersal distance after 5 days, and (**d**) distance between shelter sites and foraging areas (at 2200 h). Log back-transformed means ± SEs; different letters indicate significant differences (*p* < 0.05), whereas marginal effects (*p* < 0.10) are indicated with italics. The map shows the location of these tracking sites. Toads from French Guiana were originally exported from Cayenne, visible 8 km NW of Montjoly. Its position is indicated with an arrow on the inset map of South America. See Supplementary Materials for extended version of this caption. Photo credit: J. DeVore.

**Figure 5:**
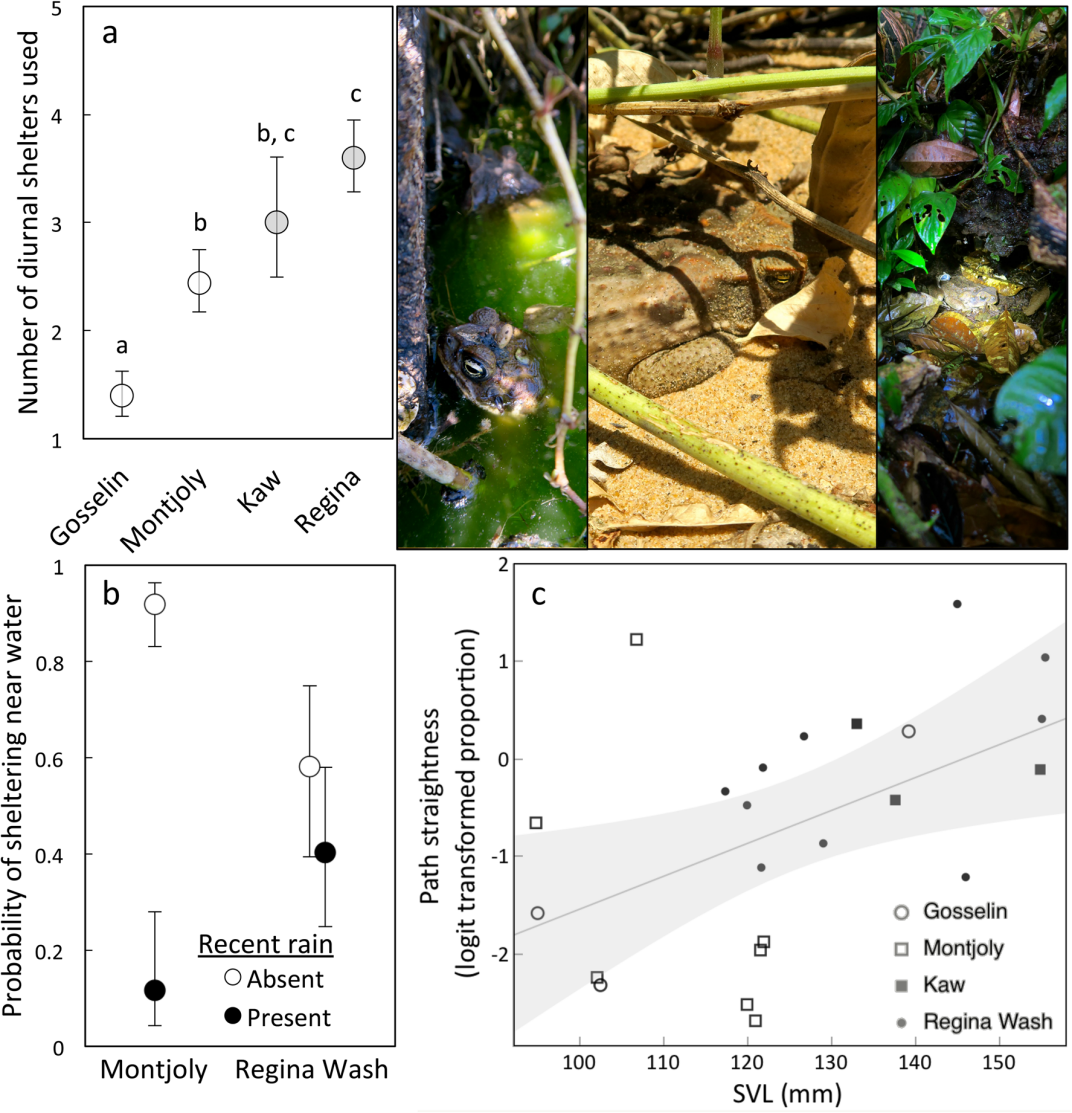
Effects of site, rainfall and toad phenotype on diurnal shelter selection: (**a**) number of shelters used, (**b**) probability of sheltering near water, (**c**) path straightness. Graphs show means ± SEs with different letters indicating significant differences (*p* < 0.05; **a, b**) or linear regressions with 95% CI (**c**). The images show coastal toads on the left, either sheltering in the rock pool at Montjoly or on the beach. On the right, a toad from Kaw is shown sheltering under a log in the rainforest. See Supplementary Materials for extended version of this caption. Photo credit: J. DeVore.

The probability that a toad would change shelter sites varied among sites (χ^2^ = 19.92, df = 3, *p* = 0.00018; Fig. 4), such that toads at Gosselin Beach were less likely to change shelters than were toads at any other site (*p* < 0.05). Montjoly Beach toads were also marginally less likely to change shelters than were toads at Regina Wash (*p* = 0.0528). There was also an effect of site on the distance between shelter sites (F_3,20_ = 9.68, *p* < 0.0001; Fig. 4); when toads did change shelter locations, toads at Montjoly moved farther to reach their new shelter than did toads at either Kaw (*p* = 0.0164) or Regina Wash (*p* = 0.0002). As a result, the distances moved between days also varied by site (F_1,3_ = 3.185, *p* = 0.0385); toads at Gosselin moved less than toads at Regina Wash (*p* = 0.0369) and tended to move less than those at Montjoly (*p* = 0.0940). The number of independent shelter sites a toad used over the 5-day period also varied between sites (LR χ^2^ = 33.74, df = 3, *p* < 0.0001; Fig. 5), with toads from Gosselin using fewer shelters than toads at any other site (*p* < 0.05) and toads from Montjoly using fewer shelters than those from Regina Wash (*p* = 0.0474). However, the odds that a toad would shelter near water did not vary significantly among sites (χ^2^ = 5.40, df = 3, *p* = 0.145). Total dispersal distance by the conclusion of the study period (i.e., the distance between the Day 1 and Day 5 shelters) also differed among sites (F_3,28_ = 4.98, *p* = 0.0068); toads at Gosselin did not disperse as far as those at Regina Wash (*p* = 0.010) and tended to disperse less than those at Kaw (*p* = 0.0559). However, path straightness did not vary significantly among sites (F_3,20_ = 2.01, *p* = 0.145).

Foraging movements also varied by site. Although there was no significant effect of site on the probability that toads would emerge to forage (χ^2^ = 2.73, df = 3, *p* = 0.435) or distance from the diurnal shelter (F_3,29_ = 0.543, *p* = 0.656), foraging distances did vary between sites (F_3,28_ = 3.78, *p* = 0.0214; Fig. 4). When toads did emerge to forage, Montjoly toads travelled farther to reach their foraging location than those at Regina Wash (*p* = 0.0098) and tended to travel farther than toads at Gosselin (*p* = 0.095). There was also an effect of time on foraging position; toads located later in the night were less likely to be outside of their diurnal shelter (odds ratio per hour = 0.437, SE = 0.329 to 0.580, χ^2^ = 8.98, df = 1, *p* = 0.0003), were closer to their diurnal shelters than were toads located earlier in the night (F_1,66_ = 7.02, *p* = 0.010), and had reduced foraging distances (effect size per hour = − 0.148 m, SE = − 0.210 to − 0.081 m; F_1,53_ = 4.47, *p* = 0.0392).

### Effects of rain on toad movement

The type of diurnal shelter site a toad selected (i.e., near or away from water) was affected by a significant interaction between site and rainfall (site*rain: χ^2^ = 7.93, df = 1, *p* = 0.005; rain: χ^2^ = 9.73, df = 1, *p* = 0.0018; site: χ^2^ = 0.495, df = 1, *p* = 0.482). At Montjoly, toads were less likely to shelter near water following rain, such that the relative odds of sheltering near water when it had not rained were 86.6 times those when it rained (SE = 30.2 to 248.4, df = 36, t = − 4.24, *p* = 0.0002). At Regina Wash, rain did not affect shelter preference (odds ratio = 2.06, SE = 1.11–3.83, df = 39, t = − 1.17, *p* = 0.249; Fig. 5). However, note that standing water was also more available at Regina Wash than at Montjoly (fresh/brackish water covered ~ 2% of the convex polygon encompassing all toad positions at Regina Wash [~ 350 m^2^ of 16,000 m^2^] vs. < 0.1% at Montjoly [~ 30 m^2^ of 67,000 m^2^]). We excluded Kaw from this analysis due to the low sample size combined with a lack of variation in toad position, which prevented a good model fit. (At Kaw, two toads consistently sheltered away from water. The remaining toad spent 3 days in a burrow beside the pond before moving into forested habitat for the 2 days when it had rained.) In contrast, rain did not significantly affect the distance moved between days, the probability of changing diurnal shelters, or the distance between consecutive shelter sites, nor were there significant interactions between site and rainfall for these variables (*p* > 0.1 in all cases). Rain did not significantly affect nocturnal positions at Regina Wash (the only site where nocturnal sampling was conducted following rain; *p* > 0.1 in all cases).

### Variation between individuals and its effects on movement

**Figure 6:**
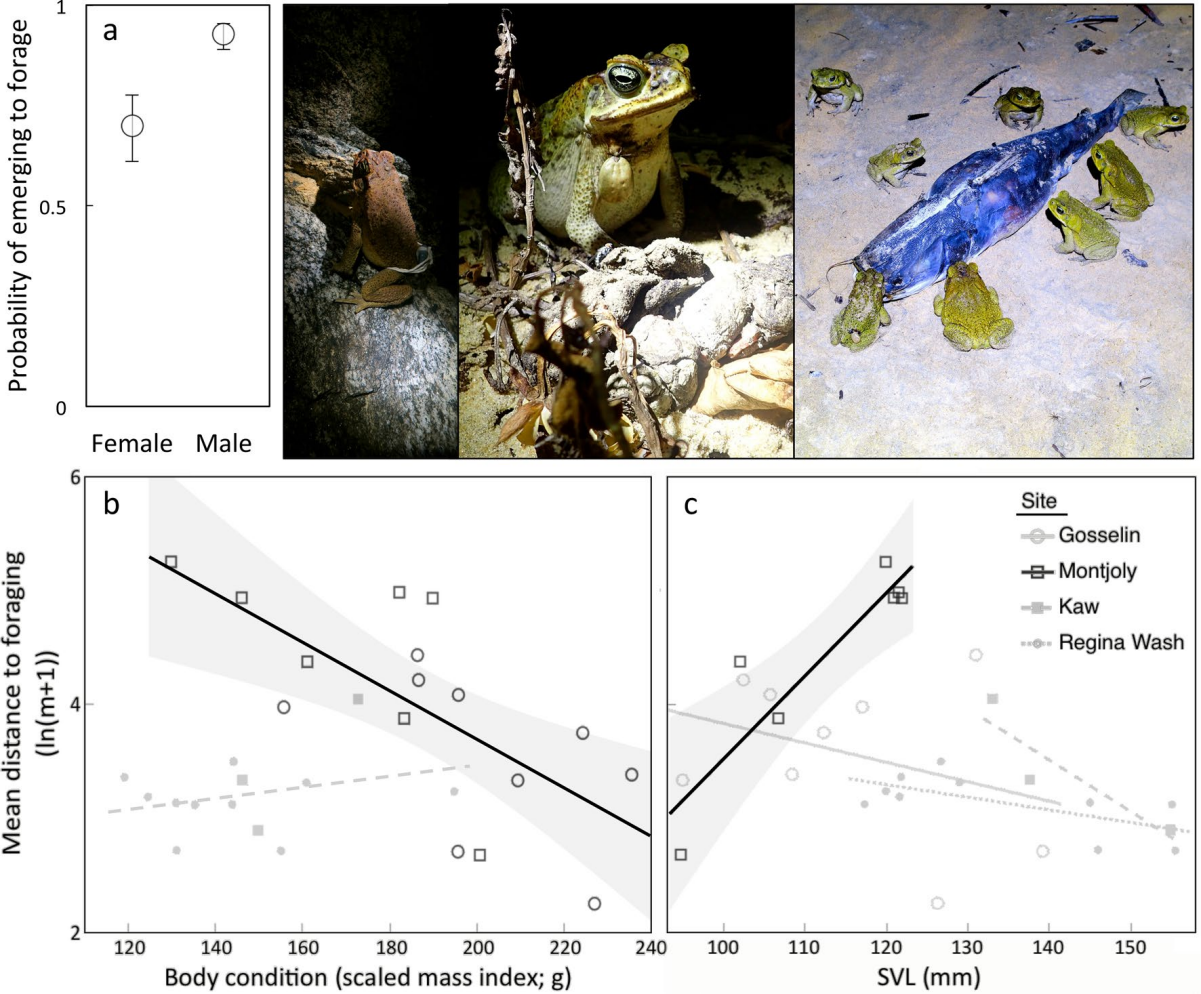
Foraging behaviour as a function of sex, body condition and/or snout-vent length, showing: (**a**) the probability of emerging to forage (means ± SEs), and distances to foraging sites from shelter sites as a function of body condition (**b**) or snout-vent length (**c**). Significant regressions depicted with 95% CI (**b, c**). The effect of body condition was consistent across sites of the same habitat type, so a single regression line per habitat type is shown in panel (**b**) for simplicity. The photos depict toads in coastal habitats foraging, including on invertebrates around a dead fish. See Supplementary Materials for extended version of this caption. Photo credit: J. DeVore and S. Ducatez.

Because we did not detect females at our rainforest sites during the tracking period, we first analysed the effects of individual-level predictors within the coastal sites alone. This allowed us to determine whether sex affected movement by comparing toads of different sexes from the same site. The only parameter significantly affected by sex was the probability of emerging to forage; within coastal sites, the odds that a male toad would emerge were 7.37 times those of a female (SE = 2.69 to 20.16, χ^2^ = 3.93, df = 1, *p* = 0.0473). There were no significant effects of site, SVL, or body condition on these odds. Once coastal toads had emerged, the distance they travelled was negatively associated with their body condition (F_1,11_ = 13.84, *p* = 0.0034), with toads in poor body condition travelling farther during their foraging expeditions (Fig. 6). This distance was also affected by a significant interaction between site and snout-vent length (SVL; F_1,11_ = 11.34, *p* = 0.0063); larger toads travelled farther from their diurnal shelters to forage at Montjoly, but not at Gosselin (Fig. 6). The only individual-level predictor of nocturnal distance from the diurnal shelter was body condition, with toads in poorer body condition found farther from their diurnal shelters across both coastal sites (effect size per g = − 0.0187 m, SE = − 0.0271 to − 0.0103 m, F_1,14_ = 5.25, *p* = 0.0380). There was also a significant interaction between time and site in this model (F_1,35_ = 5.42, *p* = 0.0258); toads were found farther from their diurnal shelters earlier in the night at Montjoly (*p* = 0.031), but there was no effect of time on nocturnal position at Gosselin (*p* = 0.829). In contrast to these nocturnal movements, which were apparently affected by variation among individuals, the only factor that significantly influenced coastal movements between days and the probability of changing shelters was site (see previous section for model outputs), although coastal toads in poorer body condition tended to travel farther when they moved to a new diurnal shelter (F_1,8_ = 4.12, *p* = 0.0768; effect size per g = − 0.0191 m, SE = − 0.0284 to − 0.0098 m).

When potential effects of individual variation on the probability of emerging to forage were considered across all four sites, sex was the only significant predictor; overall, the odds that a male would emerge to forage were 5.48 times those of a female (SE = 2.98 to 10.11, LR χ^2^ = 8.16, df = 1, *p* = 0.004). To avoid confounding the influences of site and sex, we omitted sex from all models in which no sex effect had been detected at coastal sites (i.e., all subsequent models). Across sites, the distance travelled by toads that had emerged to forage was affected by a significant interaction between site and SVL (F_3,17_ = 4.92, *p* = 0.0122), as well as site and body condition (F_3,17_ = 4.28, *p* = 0.020). Unlike at coastal sites (see above), there were no significant effects of size or body condition on foraging distance at rainforest sites (*p* > 0.1; Fig. 6). Time was also a significant predictor in this model; toads captured later were closer to their diurnal shelters (F_1,51_ = 4.47, *p* = 0.0394). Distance from the diurnal shelter was not related to any of the individual-level characteristics measured (*p* > 0.1 in all cases).

The probability that a toad would change diurnal shelters was affected by both SVL (LR χ^2^ = 7.99, df = 1, *p* = 0.005) and site (LR χ^2^ = 41.34, df = 3, *p* < 0.0001). Larger toads were less likely to move to a new shelter (odds ratio per mm = 0.950, SE = 0.932 to 0.968), toads at Gosselin were less likely to change shelters than were toads at any other site (*p* < 0.05), and toads at Montjoly were less likely to change shelters than were toads at Regina Wash (*p* = 0.0029). However, no individual-level predictors were significantly associated with the distance between consecutive diurnal shelters, leaving only the site effect in the final model (Fig. 4b). The distance between days was also affected by both SVL (F_1,25_ = 8.62, *p* = 0.007) and site (F_1,25_ = 5.50, *p* = 0.0048), with smaller toads travelling farther between days (estimate per mm = − 0.0344 m, SE = − 0.0458 to − 0.0228 m) and toads at Gosselin staying closer to their previous shelter than did toads at any other site (*p* < 0.05 in all cases). Path straightness was also associated with SVL (F_1,21_ = 6.98, *p* = 0.0153); the movements of larger toads were straighter (Fig. 5). Total dispersal distance over the study period was affected by both body condition (F_1,25_ = 6.187, *p* = 0.0199) and site (F_3,25_ = 8.67, *p* = 0.0004). Toads in better body condition dispersed farther (effect per g = 0.0262 m, SE = 0.0156 to 0.0370 m). When body condition was included in the model, dispersal was significantly lower at both of the coastal sites than either rainforest site (*p* < 0.05 for all comparisons), but there was no significant difference between sites of the same habitat type (*p* > 0.2 for both coastal and rainforest). However, note that we lacked data on body size for the toad that dispersed the farthest at Montjoly (M7; 424 m), so this toad was excluded from models that included body condition.

## Discussion

Broadly, activity patterns of cane toads in their native range resemble those in the areas to which this species has been translocated. Adult toads are largely inactive by day, although occasional individuals forage during daylight hours^51,52^, or leave their diurnal shelter sites to escape lethally high temperatures^53^. Our observations of diurnal activity in radio-tracked toads at the French Guiana study sites (in 4 of the 34 toads^51^) indicate that diurnal foraging sometimes occurs within the native range as well as invaded regions, though such behaviour is relatively rare. Overall, movements varied among sites as well as among individuals, indicating that toads exhibit substantial behavioural flexibility in their native range; these toads not only adjusted their movement patterns in response to environmental conditions, but also exhibited environment-dependent flexibility in response to individual-level variation in morphology or state.

Although our toads were generally sedentary by day, they regularly emerged after nightfall and moved to places where they could access resources such as food, water, or mates. Male toads were more likely to emerge, which may partially reflect differences in breeding behaviour; adult males can spend long periods in waterside habitats (requiring migration to and from the pond) whereas females visit ponds less often (and generally, to rehydrate rather than to breed^54,55^). For example, a trend for males to move further at night was seen in toads spool-tracked in Hawai’i^27^, where males travelled farther than females at certain sites in order to reach their breeding ponds. (However, studies in Australia have not reported significant sex differences on the probability of emerging or nocturnal movements^45^). Ultimately, these nocturnal foraging excursions took the animals up to 200 m from their diurnal retreats, with foraging toads showing a mean displacement of 56.4 m (SE = 5.8 m; 48.8 ± 5.3 m if toads that did not emerge are included). These nocturnal movements are similar to those recorded in their invasive range in Hawai’i, where toads were found to have a mean displacement from their diurnal shelter of 27.6 m (SE = 10.4 m; range 1–300 m)^27^.

However, foraging behaviour also varied among study sites. Inspection of the routes travelled by toads suggests that these foraging routes depend on local topography, and the spatial location of shelter sites versus opportunities for foraging and hydration. For example, coastal habitats imposed a strong spatial separation between the availability of water for hydration and access to prey. This segregation was especially evident at Montjoly, where on all occasions that toads foraged on the beach, they were > 100 m from the brackish-water rock pool frequently used as a diurnal shelter site by rehydrating toads. Marine subsidies appear to be critical food resources for toads in coastal habitats (e.g., edible insects attracted to dead fish or broken/hatched sea turtle eggs; see Fig. 6). In order to exploit this mosaic of profitable but spatially separated resources, toads have to move long distances between moist shelter sites and foraging areas; as toad densities around limited water resources increase this distance may increase further in response to intraspecific competition^56^. In contrast, toads in rainforest sites were not restricted by water limitation when selecting their diurnal shelters, did not shelter together in groups, and would not increase their access to prey by moving long distances to and from their diurnal retreats. Indeed, prey distribution in these environments may be better suited to the sit-and wait foraging strategy commonly used by toads than the active, more energetically demanding strategies favoured by a patchy distribution of ephemeral but abundant prey^43^. As a result, rainforest animals did not travel far from their diurnal shelters at night (mean = 32 m, SE = 5 m; Fig. 4). In short, cane toads within their native range appear to flexibly adjust their movement patterns to the spatial location of critical resources within the landscape. Such ecological flexibility may be a key predictor of success if the species is translocated to a new country^57^.

Foraging movements were also affected by the body condition of individual toads, with coastal toads in poor body condition travelling farther during their foraging expeditions than healthier toads. Such state-dependent foraging decisions occur in a wide variety of species^43,58–60^. Here, hunger may drive these individuals to forage more extensively^43^, despite desiccation risk in exposed beach habitats. The link between foraging behaviour and body condition in coastal environments but not rainforest environments may reflect the apparent differences in prey distribution/abundance, predation risk, and the anti-predator strategies that toads utilized in these habitat types^43^, but further research is needed to understand how selection pressures modify toad responses to resource depletion in these different environments.

Because toads often move extensively at night, studies that rely upon locating toads during daylight hours only, when the animals are ensconced in their retreats, can substantially underestimate the total distances moved by individuals on a daily or weekly basis. However, daily checks of shelter sites are well-suited to measuring overall rates of displacement, a critical variable for determining rates of geographic spread in invasive populations^17^. These daily movements are also influenced by individual phenotype. Whereas smaller toads in our study were more likely to change shelter sites and moved more between days (a trend also observed in some Australian populations^19^), when larger individuals moved, they did so in straighter paths—that is, a more consistent direction from one day to the next. Interestingly, studies on invasive toads in Australia have revealed inter-population (geographic) differences in path straightness, and significant heritability of this trait^13^. Hence, a trait that is linked to body size within the native range has evolved to differ geographically (but not related to body size) within the species’ invasive range. That evolved shift contributes to the faster dispersal of invasive toads within Australia, a target of selection^61^, suggesting that evolution has taken a dispersal-relevant trait (path straightness) that showed pre-existing variation, and changed the distribution of that trait among rather than within populations. Such a decoupling of pre-existing variation to morphology may provide a rapid way for selection to generate novel trait distributions.

Weather can also have pronounced effects on amphibian movements. In Australia, cane toads exhibit nomadic behaviour during the wet season, and greater fidelity to both their diurnal shelters and their site during the dry season^12,32,45^. This shift is associated with increased desiccation risk during the dry season, coupled with greater predation risk and lower prey availability, all of which increase the risk of moving at this time^45^. Similarly, toads in the Australian semi-desert sheltering away from water move rapidly through the landscape along relatively straight paths following rain, likely to increase their probability of encountering standing water (mean straightness index = 0.642, SE = 0.075). In contrast, semi-desert toads sheltering near water bodies are more mobile during dry periods, but move along meandering paths (mean straightness index = 0.416, SE = 0.078^19^). Although seasonality is more extreme in the Australian savannah than in French Guiana^12^, rainfall may also affect toad movements at our coastal sites. Coastal toads were more likely to reuse shelters than were toads in the rainforest, and at Montjoly, where the preferred foraging area was > 100 m from the rock pool typically used for rehydration, rain had a pronounced effect on where toads sheltered (Fig. 5). For example, four toads that had never sheltered away from the rock pool moved to diurnal shelters on the beach following rain (50–320 m away) before returning to the rock pool the following day. In fact, the largest movements we recorded between consecutive shelters (320 m and 250 m) were both associated with this rain event at Montjoly, with the six Montjoly toads that changed shelters following this rain event moving, on average, 170.5 m to reach their new shelter (SE = 41.5 m). This response to rain highlights the likelihood that coastal toad movements were restricted during the tracking period, such that the lack of alternative water sources forced these toads to return to the same diurnal shelter (resulting in path straightness measurements at coastal sites somewhat lower than those observed near waterbodies in the Australian semi-desert; coastal mean straightness index = 0.238, SE = 0.112). Sand beaches drain rapidly, leaving little soil moisture, and the month when the coastal toads were tracked was exceptionally dry (5 mm/month). This rainfall is substantially less than the average rainfall from December to July (which is consistently > 150 mm/month, with a maximum rainfall in May of 520 mm and 26 rainy days/month)^41^; the near-daily occurrence of rain at this time is likely to reduce reliance on standing water, which could allow greater dispersal than was observed during our study (for example, the abundances of toads rehydrating around waterbodies decrease during the wet season in native populations in Venezuela and Brazil^62^). In addition, although breeding occurs year-round, influxes of toads to French Guiana breeding sites occurred in December, indicating that toads may be more mobile during the wet season. This reliance on limited freshwater resources during the drier months may also explain why toads lived at such high densities at coastal sites, despite the fact that these dense populations suffered from high levels of parasitism as a result^40^. Limited rainfall during the tracking period may therefore have restricted toad movements, especially at coastal sites, where seasonality is more pronounced and water availability was limited.

The variation in toad spatial ecology among sites, and its links to toad size, body condition, and weather, make it difficult to draw simple comparisons with studies of movement ecology in the species’ invasive range. Nonetheless, our study supports a basic (but hitherto untested) premise of previous interpretations of evolved shifts in movement ecology within invasive populations of cane toads. Within their native range (and presumably reflecting the ancestral condition for toads translocated to other countries), toads tend to be sedentary, with little net displacement over a 5-day period. Indeed, the net displacement of toads at the Gosselin beach site (~ 1.5 m over 5 days) is lower than reported in most other studies. In the course of our study, most toad movements were more meandering than directional, and only one toad actually left a study site; a female toad that dispersed from the Montjoly pool to a suburban ditch 420 m away (30 days later, this female was still present in this ditch, indicating that this movement was not a short-term displacement). Toads from both coastal and rainforest habitats moved in meandering paths, such that path straightness did not differ significantly between habitat types (mean coastal straightness index = 0.416, SE = 0.078, rainforest = 0.453, SE = 0.086). Whereas coastal toad dispersal was likely restricted by a lack of water during the study period, dispersal by rainforest toads may have been restricted by their nocturnal preference for open habitats for breeding and foraging activities. The availability of such open areas is limited in dense rainforest environments, and rainforest toads frequently returned to the same forest gaps at night. (Although roads could be considered open habitats, and are often used as dispersal corridors in Australia^10,63^, we never observed toads moving along roads in French Guiana.) As a result, cane toads in our study only displaced 37.4 m (SE = 14.1 m) on average over the 5-day period, with a mean displacement between consecutive shelters of only 68.9 m (SE = 9.6 m). Although greater than the 11 m movements between consecutive shelters recorded in invasive Hawai’ian populations^27^ and similar to mean values recorded in established Australian populations (i.e., 65–90 m), this value is substantially lower than the very high dispersal rates of invasion front toads in tropical Australia, where toads move ~ 270 m between consecutive diurnal shelters (and up to 1.8 km)^17,18,20^. This discrepancy indicates that these high dispersal rates along the invasion front are indeed derived traits for the species, whereas movements in Hawai’ian toads may actually be somewhat reduced relative to those in the native range.

Our study of native-range toads has shown great flexibility in movement patterns in response to local conditions. Toads in the native range exploit a wide range of habitats, including beach and rainforest sites otherwise typified by profoundly disparate faunal groups, exhibiting a level of habitat generalism that likely facilitated this toad’s success and subsequent colonization of a wide range of habitats in their introduced range^40,62,64^. Native-range toads also exhibited strong behavioural plasticity in response to environmental conditions and utilized different strategies to exploit available resources in different habitats; such plasticity may also increase performance in species invading novel environments^65,66^. Most remarkably, we found that some toads regularly traverse long distances to exploit marine food subsidies on the open beach before returning to rehydrate in brackish-water pools (to > 4ppt; see Fig. 1 extended caption in the Supplementary Materials). This behaviour not only highlights the ability of cane toads in some populations to disperse long distances across open habitats, but also their ability to exploit xeric environments. The fact that these toads were extremely reliant on limited water resources that were susceptible to both drying and tidal inundation suggests that toads from coastal populations cope with periodic water shortages in order to persist in these environments; indeed, the behavioural strategies for reducing water loss observed in coastal toads mirror those utilized by introduced toads invading arid semi-desert habitats, surviving in seasonally dry savannah, or inhabiting lava fields (e.g., higher rates of shelter reuse, reduced path straightness, reduced dispersal, and a reliance on rainfall when moving away from waterbodies^9,12,19,27,32,45,67^). Additionally, some strategies thought to be novel behavioural adaptations to the arid environments of Australia were also observed at coastal sites; for example, whereas toads generally spend the day in terrestrial shelters, moving to water to rehydrate nocturnally as necessary, toads in semi-arid environments have also been found to rehydrate in standing water during the day (an apparent behavioural response to diurnal desiccation risk and temperature stress^53,67^). We observed similar behaviours at our coastal sites, where a high proportion of toads diurnally sheltered partially immersed in standing water, rather than in terrestrial shelters (e.g., Fig. 5). The ability of cane toads to successfully invade arid habitats has been one of the most surprising aspects of their invasion, perhaps because most information on native-range toads comes from inland populations or those living in anthropogenic habitats^27,68–70^. This raises the possibility that adaptation to coastal environments within the native range resulted in toads that were pre-adapted to frequent, long-distance dispersal across (and the utilization of) xeric environments, such as those they would subsequently encounter in Hawai’i and Australia. Given the higher densities and greater observability of toads in beach relative to forested habitats, as well as their proximity to the sailing ships used to export these toads from Cayenne, the individuals originally collected from French Guiana for translocation to sugarcane plantations in other countries were almost certainly from coastal habitats. (Indeed, at that time the cane toad was widely known as “the marine toad”.) In the course of their invasion across tropical Australia, these pre-existing abilities have been fine-tuned by evolutionary processes; e.g., the capacity for long-distance dispersal exhibited facultatively by coastal native-range toads is now expressed constitutively in invasion-front individuals within Australia^71^. Future research may usefully explore the role of local adaptation and behavioural plasticity within the native range in preparing these toads to successfully invade and exploit such extreme habitats in their introduced range.

In summary, cane toads within their native range exploit a wide range of environments, and modify their behaviours to match the risks and opportunities presented by these different habitats. This highly adaptable species may have benefited from this broad ancestral niche, enabling the cane toad to become one of the most successful invasive species in the world.

## Supplementary Information

Supplementary Information.

## Data Availability

Data is available from the Dryad Digital Repository at 10.5061/dryad.xsj3tx9fk

## References

[1] Crystal-Ornelas R, Lockwood JL (2020). Cumulative meta-analysis identifies declining but negative impacts of invasive species on richness after 20 yr. Ecology.

[2] Emery-Butcher HE, Beatty SJ, Robson BJ (2020). The impacts of invasive ecosystem engineers in freshwaters: A review. Freshw. Biol..

[3] Simberloff D (2021). Maintenance management and eradication of established aquatic invaders. Hydrobiologia.

[4] Weidlich EWA, Flórido FG, Sorrini TB, Brancalion PHS (2020). Controlling invasive plant species in ecological restoration: A global review. J. Appl. Ecol..

[5] Simberloff, D. Invasive species in Conservation biology for all (eds Sodhi, N. S. & Ehrlich, P. R.) 131–152 (Oxford University Press, 2010).

[6] Hoddle MS (2004). Restoring balance: Using exotic species to control invasive exotic species. Conserv. Biol..

[7] Turvey, N. D. *Cane toads: a tale of sugar, politics and flawed science*. (Sydney University Press, 2013).

[8] Shine, R. *Cane toad wars*. (University of California Press, 2018).

[9] Seebacher F, Alford RA (1999). Movement and microhabitat use of a terrestrial amphibian (*Bufo marinus*) on a tropical island: Seasonal variation and environmental correlates. J. Herpetol..

[10] Brown GP, Phillips BL, Webb JK, Shine R (2006). Toad on the road: Use of roads as dispersal corridors by cane toads (*Bufo marinus*) at an invasion front in tropical Australia. Biol. Conserv..

[11] Brown GP, Shilton C, Phillips BL, Shine R (2007). Invasion, stress, and spinal arthritis in cane toads. Proc. Natl. Acad. Sci..

[12] Brown GP, Kelehear C, Shine R (2011). Effects of seasonal aridity on the ecology and behaviour of invasive cane toads in the Australian wet–dry tropics. Funct. Ecol..

[13] Brown GP, Phillips BL, Shine R (2014). The straight and narrow path: the evolution of straight-line dispersal at a cane toad invasion front. Proc. R. Soc. B Biol. Sci..

[14] Brown G, Kelehear C, Pizzatto L, Shine R (2015). The impact of lungworm parasites on rates of dispersal of their anuran host, the invasive cane toad. Biol. Invasions.

[15] Phillips BL, Brown GP, Shine R (2010). Evolutionarily accelerated invasions: the rate of dispersal evolves upwards during the range advance of cane toads. J. Evol. Biol..

[16] Phillips BL, Brown GP, Travis JMJ, Shine, (2008). R. Reid’s paradox revisited: The evolution of dispersal kernels during range expansion. Am. Nat..

[17] Phillips BL, Brown GP, Webb JK, Shine R (2006). Invasion and the evolution of speed in toads. Nature.

[18] Alford RA, Brown GP, Schwarzkopf L, Phillips BL, Shine R (2009). Comparisons through time and space suggest rapid evolution of dispersal behaviour in an invasive species. Wildl. Res..

[19] Tingley R, Shine R (2011). Desiccation risk drives the spatial ecology of an invasive anuran (*Rhinella marina*) in the Australian semi-desert. PLoS ONE.

[20] Pizzatto L, Both C, Brown G, Shine R (2017). The accelerating invasion: dispersal rates of cane toads at an invasion front compared to an already-colonized location. Evol. Ecol..

[21] Lindström T, Brown GP, Sisson SA, Phillips BL, Shine R (2013). Rapid shifts in dispersal behavior on an expanding range edge. Proc. Natl. Acad. Sci..

[22] Pettit LJ, Greenlees MJ, Shine R (2016). Is the enhanced dispersal rate seen at invasion fronts a behaviourally plastic response to encountering novel ecological conditions?. Biol. Lett..

[23] Pettit L, Greenlees M, Shine R (2017). The impact of transportation and translocation on dispersal behaviour in the invasive cane toad. Oecologia.

[24] Pettit LJ, Greenlees MJ, Shine R (2016). The behavioural consequences of translocation: how do invasive cane toads (*Rhinella marina*) respond to transport and release to novel environments?. Behav. Ecol. Sociobiol..

[25] Finnerty PB, Shine R, Brown GP (2018). The costs of parasite infection: Effects of removing lungworms on performance, growth and survival of free-ranging cane toads. Funct. Ecol..

[26] Blennerhassett RA, Bell-Anderson K, Shine R, Brown GP (2019). The cost of chemical defence: the impact of toxin depletion on growth and behaviour of cane toads (*Rhinella marina*). Proc. R. Soc. B Biol. Sci..

[27] Ward-Fear G, Greenlees MJ, Shine R (2016). Toads on lava: Spatial ecology and habitat use of invasive cane toads (*Rhinella marina*) in Hawai'i. PLoS ONE.

[28] Slade RW, Moritz C (1998). Phylogeography of *Bufo marinus* from its natural and introduced ranges. Proc. R. Lond. Ser. B Biol. Sci..

[29] Acevedo AA, Lampo M, Cipriani R (2016). The cane or marine toad, *Rhinella marina* (Anura, Bufonidae): two genetically and morphologically distinct species. Zootaxa.

[30] Freeland WJ, Kerin SH (1991). Ontogenteic alteration of activity and habitat selection by *Bufo marinus*. Wildl. Res..

[31] Seebacher F, Alford RA (2002). Shelter microhabitats determine body temperature and dehydration rates of a terrestrial amphibian (*Bufo marinus*). J. Herpetol..

[32] Schwarzkopf L, Alford RA (1996). Desiccation and shelter-site use in a tropical amphibian: Comparing toads with physical models. Funct. Ecol..

[33] Heise-Pavlov SR, Longway LJ (2011). Diet and dietary selectivity of Cane Toads (*Rhinella marina*) in restoration sites: a case study in Far North Queensland, Australia. Ecol. Manag. Restor..

[34] Jørgensen CB (1997). 200 years of amphibian water economy: From Robert Townson to the present. Biol. Rev..

[35] Kosmala GK, Brown GP, Shine R, Christian K (2020). Skin resistance to water gain and loss has changed in cane toads (*Rhinella marina*) during their Australian invasion. Ecol. Evol..

[36] Hopkins GR, Brodie ED (2015). Occurrence of amphibians in saline habitats: A review and evolutionary perspective. Herpetol. Monogr..

[37] Yasumiba K, Alford RA, Schwarzkopf L (2016). Seasonal reproductive cycles of cane toads and their implications for control. Herpetologica.

[38] Brodie S, Yasumiba K, Towsey M, Roe P, Schwarzkopf L (2020). Acoustic monitoring reveals year-round calling by invasive toads in tropical Australia. Bioacoustics.

[39] Evans M, Yáber C, Hero J-M (1996). Factors influencing choice of breeding site by *Bufo marinus* in its natural habitat. Copeia.

[40] DeVore JL, Shine R, Ducatez S (2020). Urbanization and translocation disrupt the relationship between host density and parasite abundance. J. Anim. Ecol..

[41] Météo-France. *Données publiques.* https://donneespubliques.meteofrance.fr/ (2021).

[42] Kelehear C, Shine R (2019). Non-reproductive male cane toads (*Rhinella marina*) withhold sex-identifying information from their rivals. Biol. Lett..

[43] Higginson AD, Ruxton GD (2015). Foraging mode switching: the importance of prey distribution and foraging currency. Anim. Behav..

[44] Peig J, Green AJ (2009). New perspectives for estimating body condition from mass/length data: the scaled mass index as an alternative method. Oikos.

[45] Schwarzkopf L, Alford RA (2002). Nomadic movement in tropical toads. Oikos.

[46] R: A language and environment for statistical computing (R Foundation for Statistical Computing, 2020).

[47] Pinheiro, J., Bates, D., DebRoy, S., Sarkar, D. & Team, R. C. nlme: Linear and nonlinear mixed effects models. *R package version 3.1-140* (2019).

[48] Bates D, Mächler M, Bolker B, Walker S (2015). Fitting linear mixed-effects models using lme4. J. Stat. Softw..

[49] Fox, J. & Weisberg, S. *An R Companion to Applied Regression*. Third edn, (Sage, 2019).

[50] Lenth, R. Emmeans: Estimated marginal means, aka least-squares means. *R package version 1.3.2* (2019).

[51] Pettit L, Ducatez S, DeVore JL, Ward-Fear G, Shine R (2020). Diurnal activity in cane toads (*Rhinella marina*) is geographically widespread. Sci. Rep..

[52] Doody JS, McHenry CR, Rhind D, Clulow S (2019). Novel habitat causes a shift to diurnal activity in a nocturnal species. Sci. Rep..

[53] Webb JK, Letnic M, Jessop TS, Dempster T (2014). Behavioural flexibility allows an invasive vertebrate to survive in a semi-arid environment. Biol. Lett..

[54] González-Bernal E, Brown GP, Crowther MS, Shine R (2015). Sex and age differences in habitat use by invasive cane toads (*Rhinella marina*) and a native anuran (*Cyclorana australis*) in the Australian wet–dry tropics. Austral. Ecol..

[55] Lettoof DC (2018). Cane toads beneath bird rookeries: utilization of a natural disturbance by an invasive species. Curr. Zool..

[56] Biesinger Z, Bolker BM, Lindberg WJ (2011). Predicting local population distributions around a central shelter based on a predation risk-growth trade-off. Ecol. Model..

[57] Wright TF, Eberhard JR, Hobson EA, Avery ML, Russello MA (2010). Behavioral flexibility and species invasions: the adaptive flexibility hypothesis. Ethol. Ecol. Evol..

[58] Lima SL, Dill LM (1990). Behavioral decisions made under the risk of predation: a review and prospectus. Can. J. Zool..

[59] Godin J-GJ, Smith SA (1988). A fitness cost of foraging in the guppy. Nature.

[60] Heithaus MR (2007). State-dependent risk-taking by green sea turtles mediates top-down effects of tiger shark intimidation in a marine ecosystem. J. Anim. Ecol..

[61] Brown GP, Kelehear C, Shine R (2013). The early toad gets the worm: cane toads at an invasion front benefit from higher prey availability. J. Anim. Ecol..

[62] Lampo M, Bayliss P (1996). Density estimates of cane toads from native populations based on mark-recapture data. Wildl. Res..

[63] Urban MC, Phillips BL, Skelly DK, Shine R (2008). A toad more traveled: The heterogeneous invasion dynamics of cane toads in Australia. Am. Nat..

[64] Ducatez, S., Tingley, R. & Shine, R. Using species co-occurrence patterns to quantify relative habitat breadth in terrestrial vertebrates. *Ecosphere* **5**, art152, 10.1890/ES14-00332.1 (2014).

[65] Baldwin JM (1896). A new factor in evolution. Am. Nat..

[66] Sol D, Duncan RP, Blackburn TM, Cassey P, Lefebvre L (2005). Big brains, enhanced cognition, and response of birds to novel environments. Proc. Natl. Acad. Sci. USA.

[67] Letnic M, Webb JK, Jessop TS, Florance D, Dempster T (2014). Artificial water points facilitate the spread of an invasive vertebrate in arid Australia. J. Appl. Ecol..

[68] Urban MC, Phillips BL, Skelly DK, Shine R (2007). The cane toad's (*Chaunus [Bufo] marinus*) increasing ability to invade Australia is revealed by a dynamically updated range model. Proc. R. Soc. B Biol. Sci..

[69] Zug GR, Zug PB (1979). The marine toad, *Bufo marinus*: A natural history resumé of native populations. Smithsonian Contrib. Zool..

[70] Kosmala G, Christian K, Brown G, Shine R (2017). Locomotor performance of cane toads differs between native-range and invasive populations. R. Soc. Open Sci..

[71] Gruber J, Brown G, Whiting MJ, Shine R (2017). Geographic divergence in dispersal-related behaviour in cane toads from range-front versus range-core populations in Australia. Behav. Ecol. Sociobiol..

