# Supplementary material for: Spatial ecology of cane toads (Rhinella marina) in their native range: a radiotelemetric study from French Guiana
Source: Sci Rep. 2021 Jun 3;11:11817. doi: 10.1038/s41598-021-91262-8 (PMC8175754; doi:10.1038/s41598-021-91262-8)
Supplement: Supplementary file 1 — Supplementary Information. [file 41598_2021_91262_MOESM1_ESM.docx]

**Supplementary Information**

**Spatial ecology of cane toads (*Rhinella marina*) in their native range: a radiotelemetric study from French Guiana**

**Jayna L. DeVore^1^, Richard Shine^1,2^**^🖂^**, and Simon Ducatez^1,3^**

^1^School of Life and Environmental Sciences, The University of Sydney, NSW 2006, Australia

^2^Department of Biological Sciences, Macquarie University, NSW 2109, Australia

^3^Institut de Recherche pour le Développement (IRD), UMR 241 EIO (UPF, IRD, IFREMER, ILM), Papeete, Tahiti, French Polynesia

*email: rick.shine@mq.edu.au

Herein we provide more extensive details on study sites, extended captions to facilitate interpretation of the Figures in the text, and three Supplementary Figures.

**Study sites**

In French Guiana >95% of the land area is forested, but cane toads occupy a range of habitat types from dense rainforest to coastal beaches^41^. To include that variation, we tracked toads at two sites dominated by tropical rainforest, and two in coastal beach habitats. The climate in French Guiana is hot and humid year-round, with mean air temperature >25^o^C in all months and an average monthly rainfall of >250 mm from December to June^39^ (Supplementary Fig. S1 online). Our telemetry was conducted in August (Gosselin, Montjoly, Kaw) and September (Regina Wash) 2017, which are relatively dry months but typically experience frequent rain nonetheless (>70 mm/month^39^). However, August 2017 was exceptionally dry; the coastal Gosselin and Montjoly sites only received 5 mm of rain over the course of the month (a 94% reduction from the August mean), whereas the Kaw rainforest site received ~80 mm of rain (a 40% reduction from the mean). However, rainfall at the Regina Wash rainforest site was 16% above the September average (~90 mm)^39^.

**(1) Gosselin Beach (4.8907S, -52.2529W, altitude 5 m asl).**  The area surrounding the beach of Gosselin in Rémire-Montjoly is relatively undeveloped, with a narrow band of forest between the beach and a road that parallels the coast. At the centre of this beach site lies a 1500-m^2^ pool, which is surrounded by sand except where it reaches the forest, where it is bordered by tall, dense grass. This pool contained tadpoles and newly metamorphosed toads, as well as marine/brackish water organisms (e.g., blue crabs and four-eyed fish), and was separated from the ocean by a sandbar. A second pool ~250 m to the east was also used by toads but did not contain tadpoles, perhaps due to relatively high salinity (~4 ppt). The salinity of both of these pools was variable, dramatically increasing during exceptionally high tides, when they were inundated by seawater, and decreasing following large rain events. However, the pools were stable in both salinity and size for the duration of the tracking period (see Fig. 1 for measurements). Other, smaller sources of freshwater were also present (a roadside pool, a seep, and a shallow puddle below a rock outcrop). Dozens of toads were present around the beach pools at night, or foraging along the beach; a single night survey here yielded 90 toads (73 males, 8 females, and 9 juveniles). Along the beach, toad density decreased with increasing distance from these pools. The only other herpetofauna observed at this site were sea turtles (Leatherback and Olive Ridley). Six male toads and four female toads were radio-tracked at this site.

**(2) Montjoly Beach (4.9133S, -52.2599W, altitude 5 m asl).** The eastern edge of this beach (~3 km N of Gosselin) abuts a rock outcrop. Rain results in the formation of small rock pools on this outcrop, the largest of which is a 17-m^2^ rock pool 50 m from the beach. Except for a few temporary puddles, and a manmade ditch in the suburban area that backs this beach, this is the only source of freshwater at the site. Although it was stable throughout our tracking period, even this pool occasionally dries up or (during monthly high tides) floods with saltwater. Many toads (males, females, and juveniles) were found gathered around this pool, where they spend the day in crevices alongside and in the water (Supplementary Fig. S2 online). Metamorphosing toad tadpoles were also present. By night, these toads were found in or around the pond, or foraging on the rocks or along the nearby beach; a single night survey here yielded 71 toads (38 males, 17 females, 16 juveniles). Along the beach, toad density decreased with increasing distance from the rock pool. Sea turtles were the only other herpetofauna observed during the tracking period. Four male toads and six female toads were radio-tracked at this site.

**(3) Kaw Rainforest (4.6437S -52.2991W, altitude 240 m asl).** This tropical rainforest site is ~28 km S of Gosselin, and surrounds a permanent pond in the Kaw forest. One pond edge is relatively open due to a small clearing (logged >50 years ago), but the rest of the site is heavily forested. A road parallels the site, 40 m from the pond. Three male toads and one female were radio-tracked at this site. A single night survey here only yielded these three males (the female was found the following day), and only one additional adult toad was observed here (a male found 4 nights into the tracking period). However, tadpoles and newly metamorphosed toads were also present. Other herpetofauna observed during the tracking period included poison dart frogs (*Dendrobates tinctorius* and *Ameerega hahneli*)*,* Knudsen’s frog (*Leptodactylus knudseni*), and lesser treefrogs (*Dendropsophus minutus*).

**(4) Regina Wash Rainforest (4.2944S, -52.2205W, altitude 45 m asl).** This open wash (~500 m^2^) formed within the tropical rainforest as a result of sediment deposition, and is located ~30 km S of the Kaw site (15 km from the village of Regina). It is surrounded by dense tropical rainforest and contains a stream. A road splits this site in half, with large rectangular culverts that allow the stream to flow under the road (toads also pass through these culverts). The western half of the wash is wetter, and the stream is slow and swampy, forming semi-stagnant pools and hosting organisms that favour stagnant conditions (e.g., electric eels). This stream narrows and flows more quickly after passing through the culverts. Ten male toads were radio-tracked at this site. Additional males and juveniles were observed at this site during the monitoring period; a single night survey yielded 13 toads (11 males, 2 juveniles). However, only one female was ever observed at this site, and this occurred after the tracking period (during subsequent monitoring). No tadpoles were present during the monitoring period, although breeding occurred here <1 month later. Other herpetofauna present included fer-de-lance (*Bothrops atrox*), Amazon tree boa (*Corallus hortulana*), brown-banded water snake (*Helicops angulatus*), twist-necked turtle (*Platemys platycephala*), and giant gladiator treefrogs (*Boana boans).*

**Extended Figure captions**


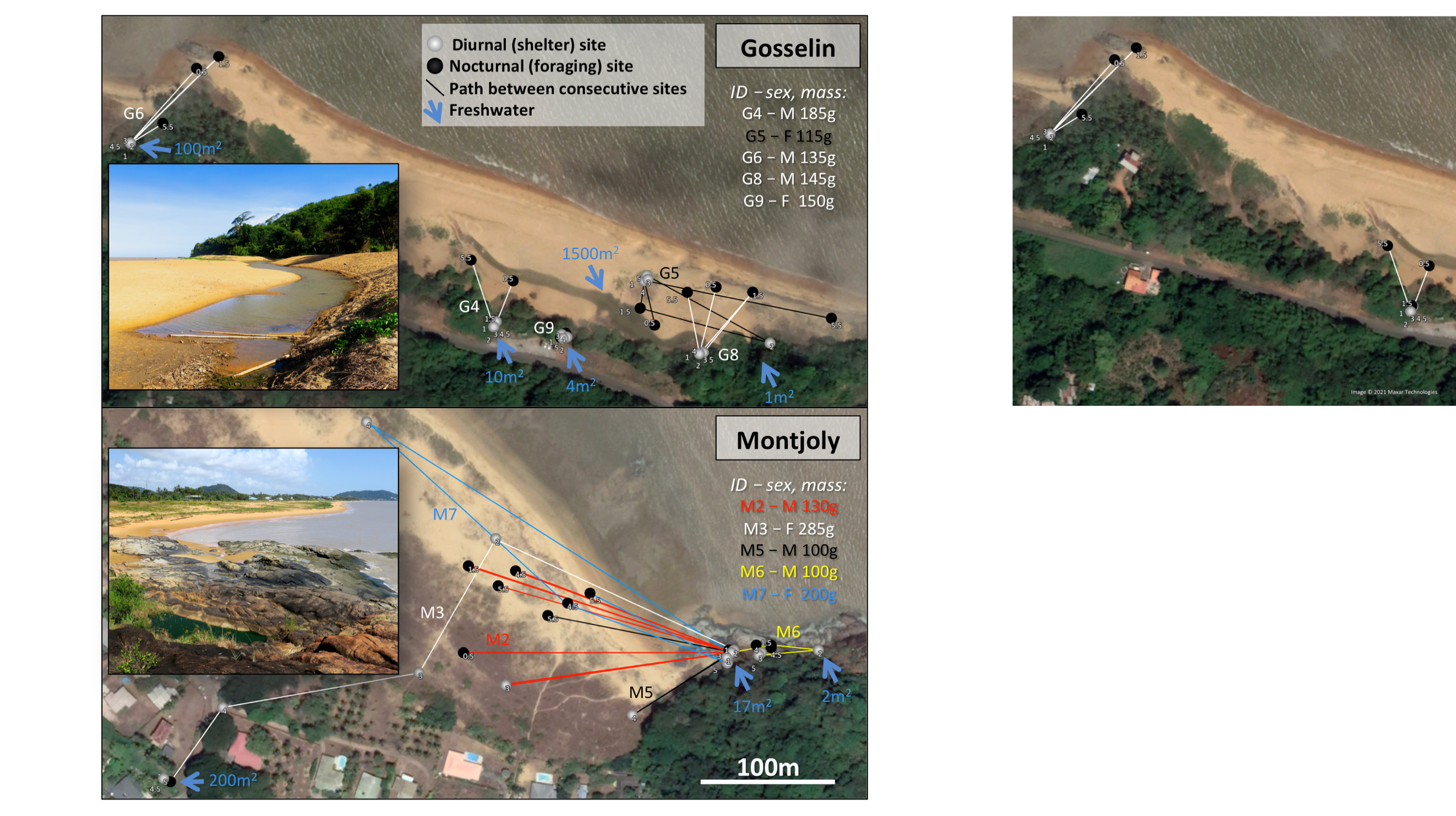


**Figure 1.** Freshwater was limited at coastal sites, and tracked toads were either consistently (e.g., G9) or periodically (e.g., M5) associated with the available waterbodies. (Note that many of the “freshwater” waterbodies used by toads for rehydration contained brackish water [i.e., >0.5 ppt]; salinities provided below.) At Gosselin these sites consisted of pools (100 m^2^, 3.66-4.65 ppt; 10 m^2^, 0.12 ppt; and 1500 m^2^, 0.15 ppt), a shallow seep (4 m^2^, 0.84 ppt), and a puddle under a low rock overhang (1 m^2^; used as the diurnal shelter of G10 [F 245 g, not shown]). Although seven of the Gosselin toads were diurnally associated with one of these five sites, the remaining three toads consistently spent their days away from water in the beach sand/vine (G5, G1 [M 265 g], G7 [M 105 g]), but were periodically present at the large 1500 m^2^ pool at night (this pool is depicted in the photo inset). Two of these toads and one seep-associated toad were also the only toads at Gosselin that ever used more than one diurnal shelter (i.e., stayed in shelters >5 m apart). At Montjoly fewer water sources were available, and all toads sheltered at the 17 m^2^ rock pool on at least one occasion (the green pool in the lower left of the photo inset, 0.74 ppt). This pool often hosted >30 toads (Supplementary Fig. S2 online). M3 was originally captured in this rock pool, but dispersed to a suburban ditch 420 m away by the end of the tracking period. Another small rock pool (2 m^2^, 0.41 ppt) was also used by one of the tracked toads on one occasion (M6); this toad foraged on the rock and was never found in the beach habitat (where all other toads were found at least once). Note that the small numbers adjacent to each point indicate the sequence in which a given individual was found at each location (e.g., 4 = diurnal location on Day 4, 4.5 = nocturnal location on Day 4). Movement data is depicted for half of the toads tracked at each site, with 5 diurnal and 3 to 4 nocturnal occasions per toad. Where tracks cross, colour-coding is used to identify individuals. Photo credit: J. DeVore, with satellite imagery from Google Earth.


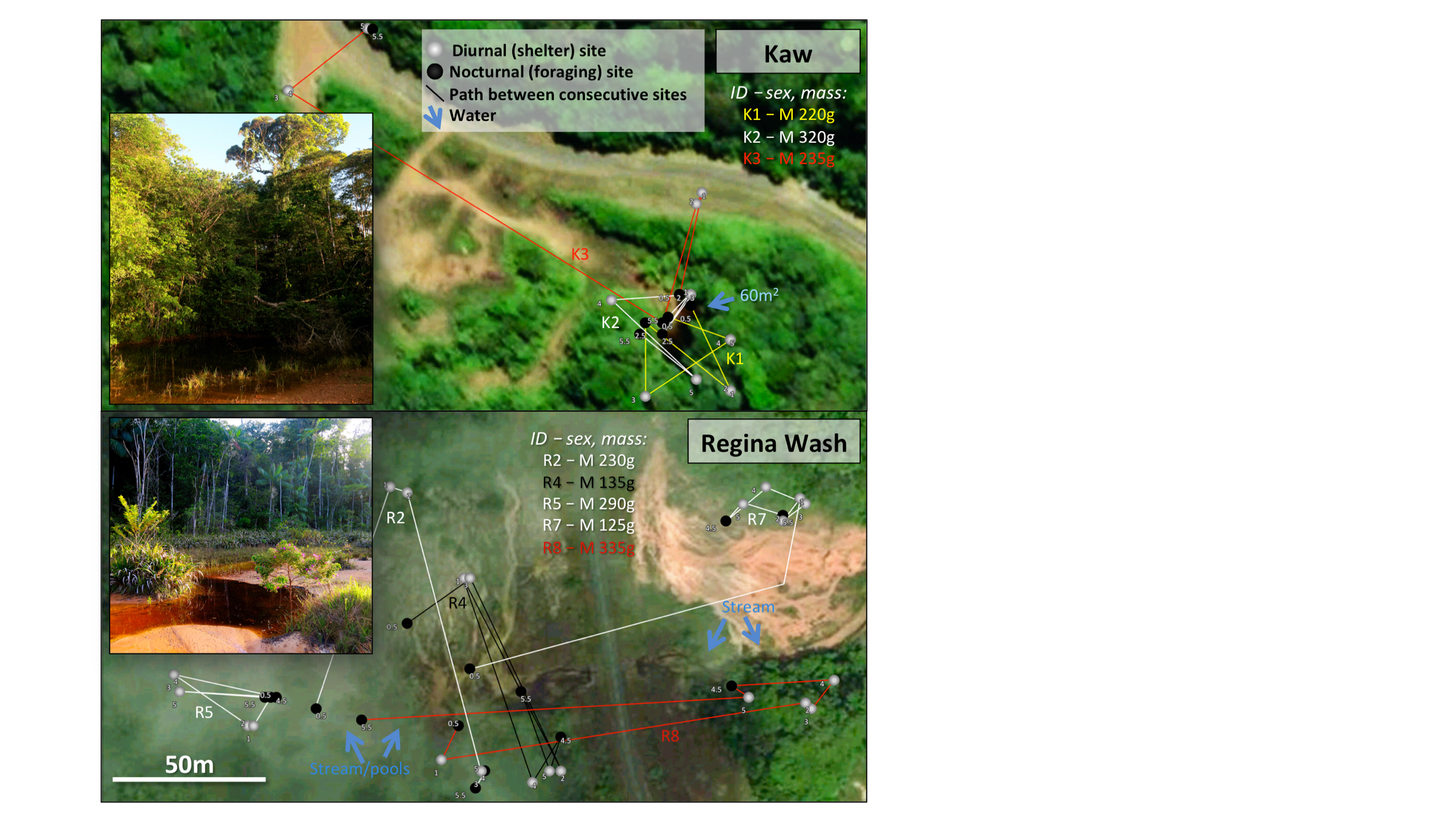


**Figure 2.** All rainforest cane toads used multiple diurnal shelters during the tracking period, with the exception of the female tracked at Kaw (not shown; K4, 690 g). K4 was always found in the same pondside burrow, other than on one nocturnal occasion, when she moved 16 m to the edge of the pond before returning to her burrow. At night, the male toads tracked at Kaw were found alongside the single breeding pond (60 m^2^) or, on one occasion, remained within their diurnal shelter (K3, Night 5). Similarly, toads tracked at Regina Wash either moved into open habitats at night, or remained in the vicinity of their diurnal shelter. However, not all of the Regina Wash toads that emerged from the forest gathered near breeding pools; toads here were instead dispersed throughout the open habitat. Note that a slow-moving stream borders the entire lower and left edges of the relatively open wash (see photo inset), becoming narrow and faster to the right. Rainforest toads generally spent the day in the forest (e.g., under or atop a log, in the leaf litter, or at the base of a plant), though some toads sheltered along the forest edge (e.g., under dense ferns, grass, or sedge), or in open habitats deep inside a burrow (R4). Note that, although all 13 male rainforest toads used multiple shelters, only one used a new shelter every day (R3; M 195 g, not shown). The remaining toads reused shelters; this reuse was not necessarily consecutive, such that some toads switched back and forth between shelter sites (Fig. 5). The small numbers adjacent to each point indicate the order in which a given individual was found at each location, with 5 diurnal and 3 nocturnal sites depicted for each toad. Movement data is depicted for three of the four toads tracked at Kaw, and five of the ten tracked at Regina Wash. Salinity was consistently <0.1 ppt in waterbodies at rainforest sites. Photo credit: J. DeVore, with satellite imagery from Apple Maps.


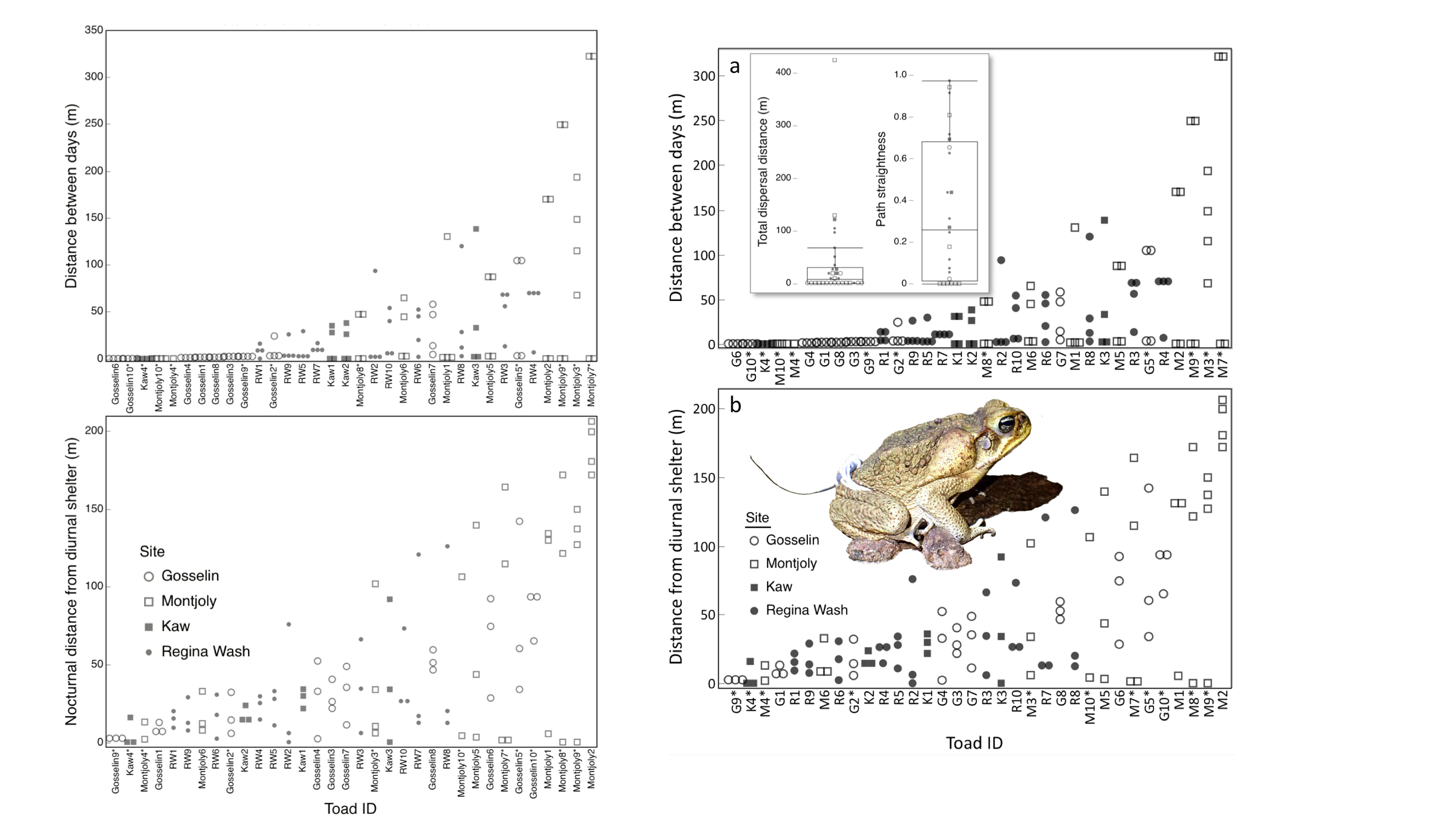


**Figure 3.** Cane toad movements varied both among individuals and across time. Here, the movements of each individual are depicted, ordered by ascending mean values. **(a)** distance each toad moved between successive days (4 values/toad, except 1 for M4). **(b)** distance toads travelled from their diurnal shelters at night (3-4 values/toad, except 2 for M4 and M10). Asterisked toad IDs identify females. The panel **(a)** inset indicates the total distance toads moved from their original diurnal shelter over the 5-day tracking period (mean: 37 m), as well as how straight this movement was (mean straightness index 0.35; a path straightness value of 0 would indicate that the toad had returned to its original shelter at the end of the tracking period, whereas a value of 1 would indicate that its movements between diurnal shelters were linear, consistently taking it farther from its original shelter – toads that never changed shelters are omitted). The photo inset in panel **(b)** shows a tracked toad from Regina Wash. Photo credit: J. DeVore.


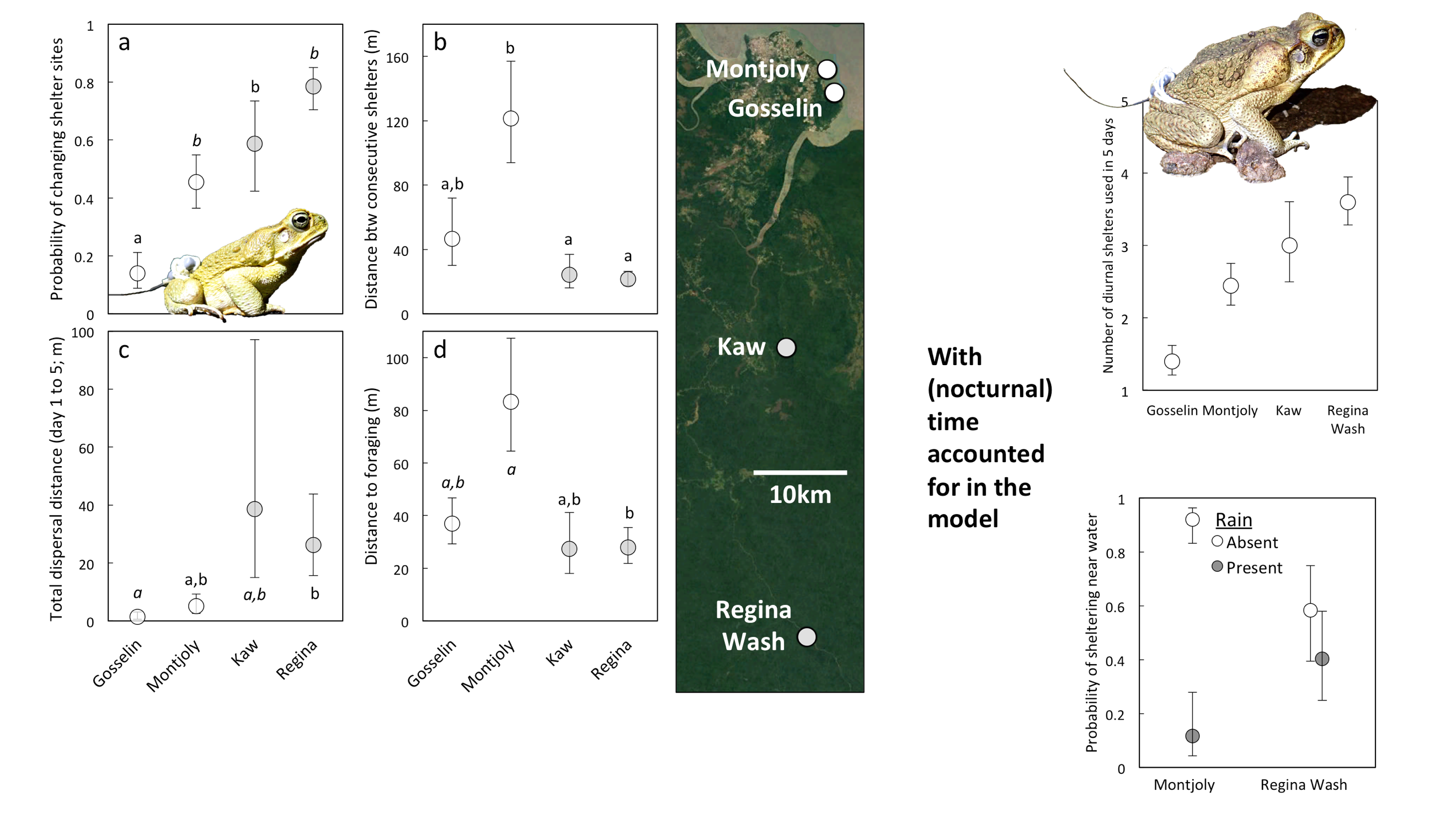


**Figure 4.** Cane toad movements over the 5-day tracking period varied between tracking sites. Toads were less likely to move to a new shelter at Gosselin than at any other site **(a)**, and toads that had left their diurnal shelter travelled farther to reach a new one at Montjoly than at either rainforest site **(b)**. Over the 5-day tracking period, toads travelled farther from their original shelter at Regina Wash than at Gosselin **(c)**. At night, toads that had emerged from their diurnal shelter travelled farther to reach their foraging location at Montjoly than at Regina Wash **(d)**; distances at 10pm. Log back-transformed means ± SE; different letters indicate significant differences (p < 0.05), whereas marginal effects (p < 0.10) are indicated with italics. White circles indicate coastal sites, whereas rainforest sites are shown in grey. The right colour panel shows the locations of these sites in French Guiana; note that the city of Cayenne (visible 8 km NW of Montjoly) is the site from which toads were exported for introduction to the Caribbean. The photo inset in panel **(a)** shows one of the tracked Montjoly males. Note that these are mean values per site, but variation between individuals also affected toad movements. Photo credit: J. DeVore.


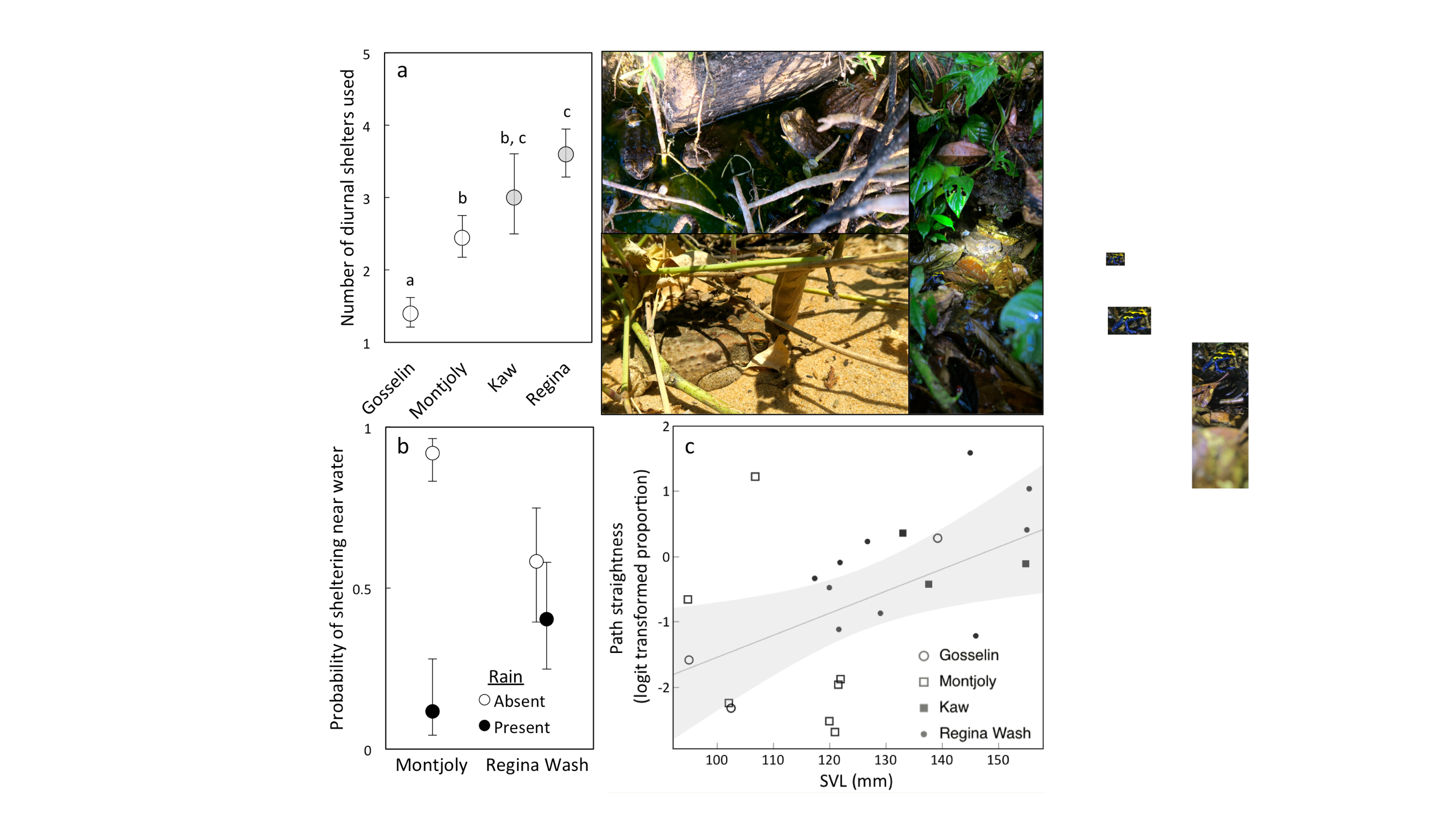


**Figure 5.** Diurnal shelter selection was influenced by site, rainfall, and phenotype. Over the 5-day tracking period, cane toads often returned to previously used shelters, but toads from rainforest sites generally used more shelters than those from beach sites **(a)**, means ± SE, different letters indicate significant differences (p < 0.05); a value of 1 would indicate that a toad used the same shelter every day, whereas 5 would indicate a new shelter every day. At Montjoly, shelter selection was strongly influenced by rainfall, with toads moving away from standing water and into beach habitats following rain **(b)**, means ± SE. At Regina Wash, there was no significant effect of rainfall on the type of shelter selected (Gosselin and Kaw are excluded from panel **(b)** due to a lack of variation). Across sites, path straightness increased with body size; larger toads meandered less in their movements **(c)**, snout-vent length (SVL), linear regression with 95% confidence intervals; straightness index was calculated as the distance between an individual’s position on the first and last day divided by the sum of the distances moved between consecutive diurnal shelters). The photo images show coastal toads on the left, either sheltering in the rock pool at Montjoly or in the sandy beach habitat. On the right, a toad from Kaw is shown sheltering under a log in the rainforest. Photo credit: J. DeVore.


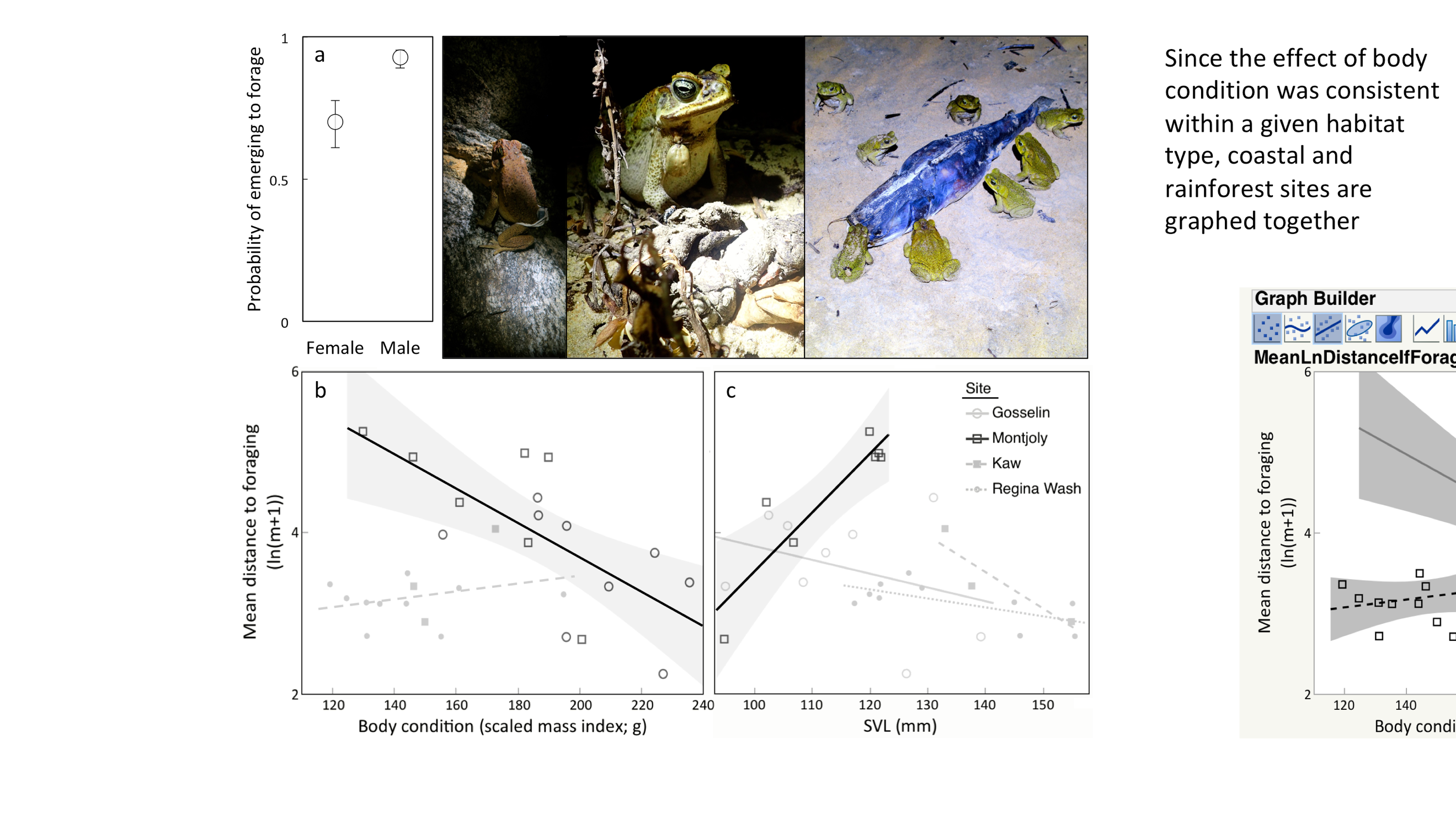


**Figure 6.** Foraging behaviour varied between sexes and, at coastal sites, as a function of body condition and/or size. Across sites, male toads were more likely to emerge to forage **(a)**, means ± SE. At coastal sites foraging toads travelled farther if they were in a poor body condition. At Montjoly, this foraging distance was also affected by size, with larger toads travelling farther. Here the mean foraging distance of each toad is depicted relative to its **(b)** body condition or **(c)** size (snout-vent length, SVL). The regression lines show the relationships between an individual’s phenotype and its foraging distance, with significant effects depicted with 95% confidence intervals. Note that the effect of body condition was consistent across sites of the same habitat type, so a single regression line per habitat type is shown in panel **(b)** for simplicity; the scaled mass index shows the mass a toad would have if all of the toads were the same length (here, 120 mm). The photos depict foraging behaviour at coastal sites, with toads foraging along rock outcrops or, more commonly, in sandy habitats. Toads in coastal habitats frequently fed on invertebrates that gathered at patchy resources, such as around dead fish or broken/hatched sea turtle eggs. Toads in rainforest habitats were never observed foraging in clusters in this way. Photo credit: J. DeVore and S. Ducatez.

**Supplementary Figure S1**

**Supplementary Figure S2**

**Supplementary Figure S3**


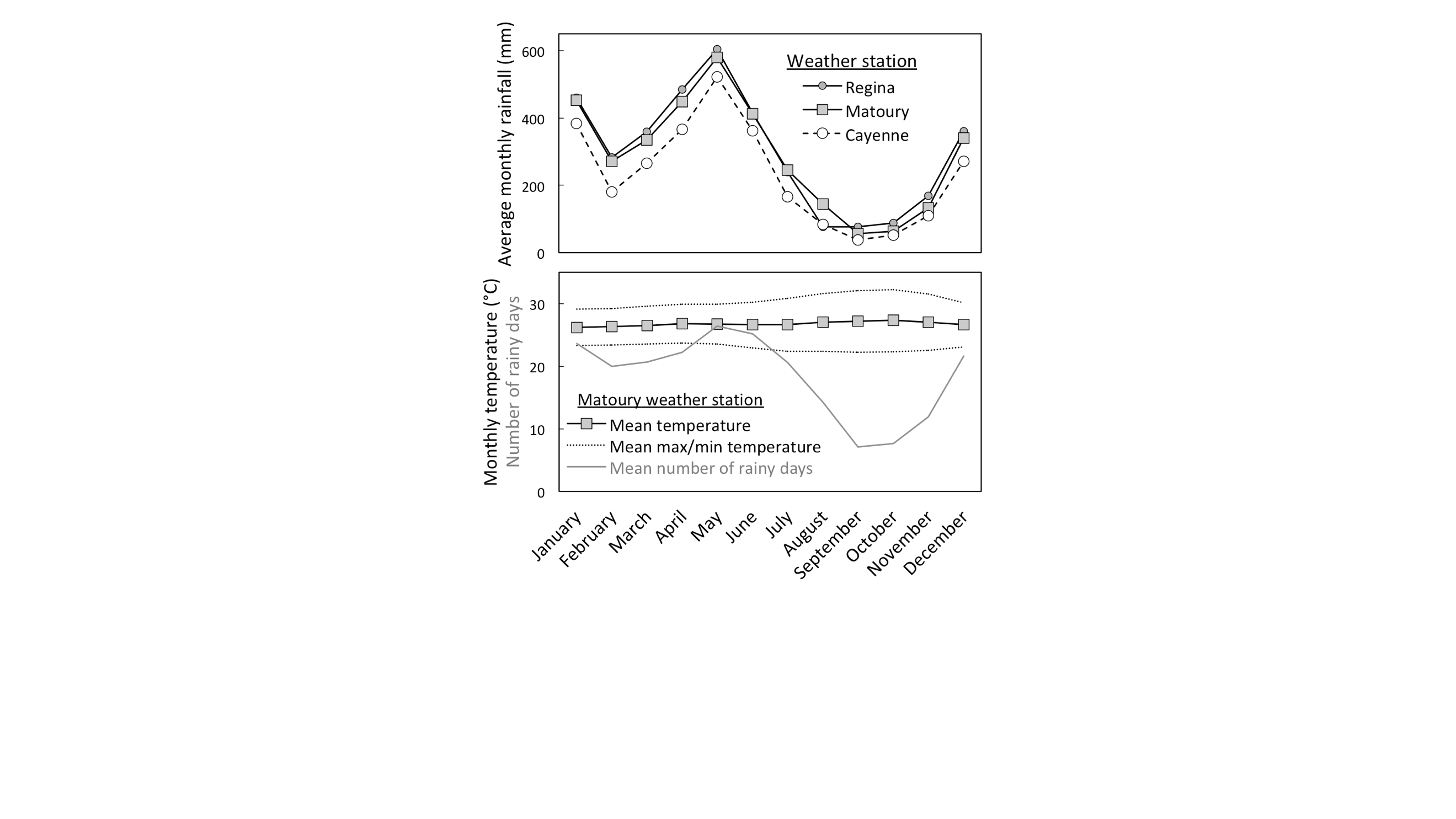


**Figure S1.** French Guiana experiences a tropical climate, with seasonal fluctuations in rainfall. Here the average monthly rainfall at the nearest weather station to each of our sites is shown (averages from 1981 to 2010^39^). The Cayenne weather station (4°56’24"N, 52°19’06"W, 4 m asl) is 7 and 9 km NW of the coastal sites at Montjoly and Gosselin, respectively. The Matoury station (4°49’18"N, 52°21’54"W, 4 m asl) is 21 km N/NW of the Kaw site (15 km SW of the Montjoly and Gosselin sites). The Regina station (4°18'51"N 52°07'52"W, 3 m asl) is 17 km E/SE of the Regina Wash site. However, note that our rainforest sites at Regina Wash and Kaw are at a higher altitude than these weather stations, which can affect rainfall at these sites (site altitudes: 45 and 240 m asl, respectively). Average monthly temperatures and the mean number of rainy days (>0.1 mm rain/day) are shown for the Matoury weather station in the lower panel. Tracking occurred in August at the Gosselin, Montjoly, and Kaw sites, and September at the Regina Wash site. Note that rainfall at the coastal sites of Gosselin and Montjoly was exceptionally low in 2017 (5 mm) relative to the August average (83 mm). This reduction in mean rainfall was substantially more pronounced along the coast (70-95% deficit) than at inland sites (30-70% deficit), such that August rainfall at the Kaw rainforest site was still 80 mm (a 40% reduction from the mean). In September, rainfall at the Regina Wash rainforest site was 16% above the average (at 90 mm). On average, temperatures were also 1°C higher than usual in August (max temp 37.5°C; Matoury mean temp: 28.1°C, avg. max: 32.4°C, avg. min: 23°C) and 1.1°C higher than usual in September (max temp 37.5°C; Matoury mean temp: 28.6°C, avg. max: 33.2°C, avg. min: 23.8°C).


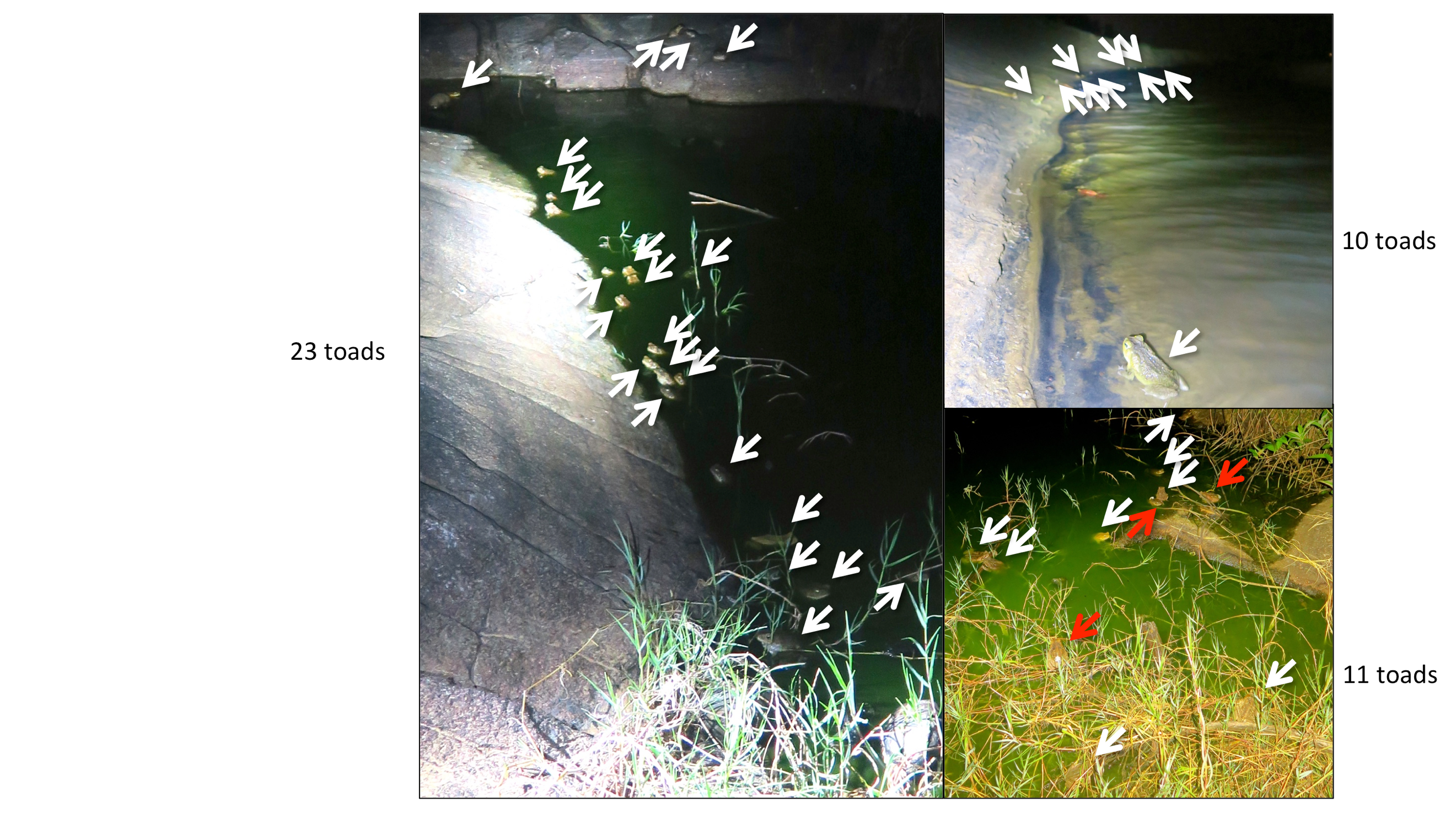


**Figure S2.** Coastal cane toads were often densely clustered around water, especially at the Montjoly site. Here the left and lower right portions of Montjoly rock pool (left and lower right panels) and the large pool at Gosselin (upper right) are depicted. The lack of alternative water sources at Montjoly resulted in most toads utilizing the same waterbody, whereas at Gosselin most of the toads gathered along the large breeding pool are males (possibly gathered for breeding purposes). White arrows indicate individual toads; toads with visible transmitters are indicated in red. Photo credit: J. DeVore and S. Ducatez.


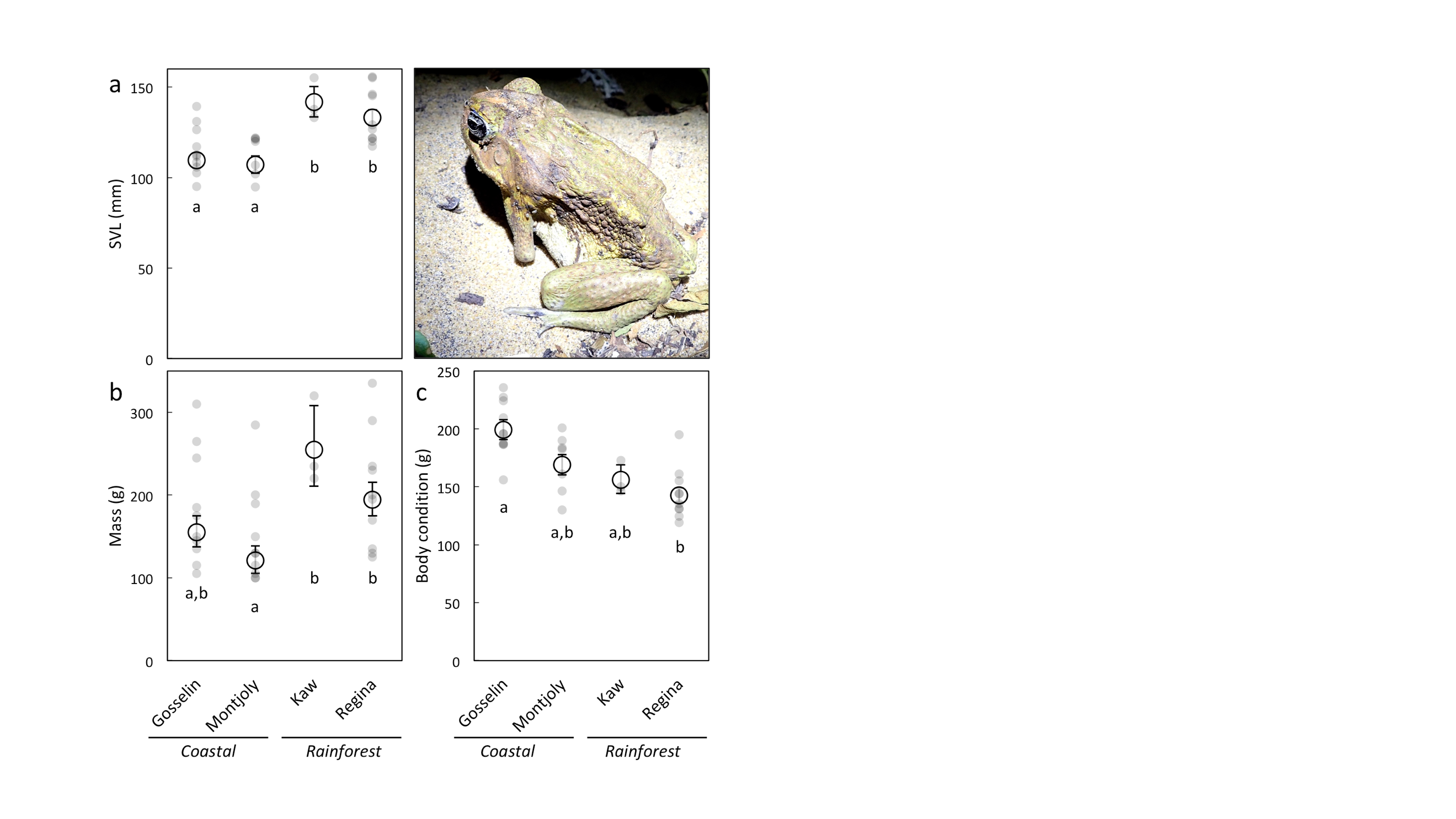


**Figure S3.** Tracked cane toads from tropical rainforest sites were significantly larger than toads from coastal sites **(a)**, snout-vent length; F_3,25_ = 7.51, p = 0.001). This size discrepancy reflected actual, pronounced morphological differences between toads from coastal and rainforest habitats (DeVore, Shine, and Ducatez unpublished data). Females were also larger than males (F_1,25_ = 4.61, p = 0.0417), as is typical for this species. The mass of the tracked toads also varied between sites (b); F_3,28_ = 2.99, p = 0.048). Females tended to be heavier, but this effect did not reach statistical significance (F_1,28_ = 3.11, p = 0.089). The body condition of the tracked toads also differed between sites **(c)**; F_3,26_ = 10.19, p = 0.0001). There was no effect of sex on body condition. There were also marginally significant differences in body condition between Gosselin and both Montjoly and Kaw, as well as between Montjoly and Regina Wash; p < 0.1 in all cases. However, note that some coastal toads were emaciated or missing limbs, such as the one pictured; emaciated coastal toads were not selected for tracking (we considered these toads to be “emaciated” if the urostyle was clearly protruding against the skin). As a result, although relatively few emaciated toads were encountered, the mean body condition of coastal toads may be lower than that of the toads selected for tracking. In contrast, nearly all of the adult toads detected at rainforest sites were also tracked (14 of 15), though one 210 mm rainforest female that was missing a leg was ultimately excluded from all analyses. Open circles depict log back-transformed means ± SEs, with different letters indicating significant differences (p < 0.05). The measurements of each individual are shown in grey. Photo credit: J. DeVore.
